# An allelic series of spontaneous *Rorb* mutant mice exhibit a gait phenotype, changes in retina morphology and behavior, and gene expression signatures associated with the unfolded protein response

**DOI:** 10.1093/g3journal/jkad131

**Published:** 2023-06-10

**Authors:** George C Murray, Jason A Bubier, Oraya J Zinder, Belinda Harris, James Clark, Mia-Cara Christopher, Courtany Hanley, Harianto Tjong, Meihong Li, Chew Yee Ngan, Laura Reinholdt, Robert W Burgess, Abigail L D Tadenev

**Affiliations:** The Jackson Laboratory, Bar Harbor, ME 04609, USA; The Graduate School of Biomedical Science and Engineering, University of Maine, Orono, ME 04469, USA; The Jackson Laboratory, Bar Harbor, ME 04609, USA; The Jackson Laboratory, Bar Harbor, ME 04609, USA; The Jackson Laboratory, Bar Harbor, ME 04609, USA; The Jackson Laboratory, Bar Harbor, ME 04609, USA; The Jackson Laboratory, Bar Harbor, ME 04609, USA; The Jackson Laboratory, Bar Harbor, ME 04609, USA; The Jackson Laboratory for Genomic Medicine, Farmington, CT 06032, USA; The Jackson Laboratory for Genomic Medicine, Farmington, CT 06032, USA; The Jackson Laboratory for Genomic Medicine, Farmington, CT 06032, USA; The Jackson Laboratory, Bar Harbor, ME 04609, USA; The Jackson Laboratory, Bar Harbor, ME 04609, USA; The Graduate School of Biomedical Science and Engineering, University of Maine, Orono, ME 04469, USA; The Jackson Laboratory, Bar Harbor, ME 04609, USA

**Keywords:** *Rorb*, allelic series mutation, mouse models, neurodevelopment, epilepsy, autism spectrum disorders, bipolar disorder, unfolded protein response

## Abstract

The *Retinoid-related orphan receptor beta* (*RORβ*) gene encodes a developmental transcription factor and has 2 predominant isoforms created through alternative first exon usage; one specific to the retina and another present more broadly in the central nervous system, particularly regions involved in sensory processing. *RORβ* belongs to the nuclear receptor family and plays important roles in cell fate specification in the retina and cortical layer formation. In mice, loss of RORβ causes disorganized retina layers, postnatal degeneration, and production of immature cone photoreceptors. Hyperflexion or “high-stepping” of rear limbs caused by reduced presynaptic inhibition by *Rorb*-expressing inhibitory interneurons of the spinal cord is evident in RORβ-deficient mice. *RORβ* variants in patients are associated with susceptibility to various neurodevelopmental conditions, primarily generalized epilepsies, but including intellectual disability, bipolar, and autism spectrum disorders. The mechanisms by which *RORβ* variants confer susceptibility to these neurodevelopmental disorders are unknown but may involve aberrant neural circuit formation and hyperexcitability during development. Here we report an allelic series in 5 strains of spontaneous *Rorb* mutant mice with a high-stepping gait phenotype. We show retinal abnormalities in a subset of these mutants and demonstrate significant differences in various behavioral phenotypes related to cognition. Gene expression analyses in all 5 mutants reveal a shared over-representation of the unfolded protein response and pathways related to endoplasmic reticulum stress, suggesting a possible mechanism of susceptibility relevant to patients.

## Introduction

*Retinoid-related orphan receptor beta* (*RORβ*) encodes a transcription factor that belongs to the nuclear receptor family along with RORα and RORγ (Becker-Andre *et al.* 1993; Carlberg *et al.* 1994; Giguere *et al.* 1994; Hirose *et al.* 1994). The *ROR*β locus maps to human chromosome 9q22 and mouse chromosome 19 (GRCm39 Ensembl release 109) (Andre, Conquet *et al.* 1998). The *RORβ* gene uses alternative first exons to produce 2 transcripts encoding RORβ1 and RORβ2 (Andre, Gawlas *et al.* 1998; Liu *et al.* 2013). *Rorb1* is expressed throughout the central nervous system (CNS) of rats in regions associated with sensory processing and circadian behavior while *Rorb2* is expressed exclusively in the eye and pineal gland where it exhibits a strong circadian expression pattern (Baler *et al.* 1996; Schaeren-Wiemers *et al.* 1997; Andre, Gawlas *et al.* 1998). Expression of both *Rorb* isoforms is regulated by the developmental stage in the retina of mice, with *Rorb1* expression peaking embryonically and *Rorb2* gradually increasing to plateau by P15 (Liu *et al.* 2013).

Much of what is known about RORβ function comes from studies in rodents. *Rorb*-knockout mice exhibit a duck-like gait, retinal degeneration, temporary male infertility, and a slightly extended period under constant darkness (Andre, Conquet *et al.* 1998). The duck-like gait phenotype may be explained by degraded sensory transmission of RORβ−positive interneurons in the dorsal spinal cord that restrict flexor activity during locomotion. Ablating RORβ interneurons of the spinal cord, and blocking neurotransmission of RORβ interneurons, were both sufficient to phenocopy the duck-like gait of *Rorb-*knockout mice and reduce primary afferent depolarization, suggesting that motor neurons are activated by lower-intensity stimulation in these mice, leading to hyperflexion (Del Barrio *et al.* 2013; Koch *et al.* 2017). Evidence from *sauteur d’Alfort* rabbits with a dramatic locomotor phenotype provides further insight regarding the cellular basis of gait deficits due to RORβ dysfunction. These rabbits contain a splice-site mutation in an evolutionarily conserved site near exon 9 of *RORβ* and produce several aberrant transcript isoforms incorporating an intronic sequence. Drastic reduction of RORβ-positive neurons in the spinal cord, and defects in the differentiation of spinal interneuron populations, are described in these rabbits, which lift their hindlimbs entirely off the ground when moving quickly (Carneiro *et al.* 2021).

The role of RORβ in development and cellular differentiation is not limited to the spinal cord. There is considerable evidence that RORβ directs cell fate in the retina. During development, RORβ is highly expressed in, and specific to, the developing retina beginning at the optic cup stage, E12.5, where it is co-expressed with other transcription factors involved in optic progenitor cell proliferation (Chow *et al.* 1998). During photoreceptor development, RORβ binds to *Opn1sw* to induce S-opsin (short wavelength, blue) expression, which is reduced in the retinas of RORβ-knockout mice (Srinivas *et al.* 2006). RORβ-knockout mice also lose rods and overproduce primitive S-cones through interactions with a rod-development pathway involving induction of *neural retinal leucine zipper* (*Nrl*) (Jia *et al.* 2009). Indeed, deletion of either *Rorb1* or *Rorb2* increases the ratio of cones to rods by 2-fold, and *Rorb2* expression is lost in *Nrl*^−/−^ mice, suggesting a reciprocal feedback mechanism that may support rod differentiation in healthy mice (Fu *et al.* 2014). Alternatively, *Rorb1-*deficient mice created by gfp cassette insertion express *Rorb2* but lose horizontal and amacrine cells in the retina (Liu *et al.* 2013).

There is also evidence that *Rorb1* functions in the developing brain. During embryonic development, *Rorb* is expressed in the neocortex of mice, but over the first postnatal week becomes highly restricted to layers IV and V of sensory areas (Nakagawa and O'Leary 2003). Indeed, RORβ and BRN1/2, 2 mutually repressive transcription factors, both function in layer IV and layer II/III patterning, respectively, where RORβ suppresses layer II/III characteristics (Oishi *et al.* 2016). Loss of RORβ function reduces the size of individual barrels in the somatosensory cortex, causes a delay in excitatory input to cortical barrels, and disrupts gene expression in layer IV neurons causing them to downregulate layer IV genes and upregulate layer V genes (Moreno-Juan *et al.* 2017; Clark *et al.* 2020). Alternatively, *Rorb* overexpression in the developing cortex of mice is sufficient to induce clustering of neurons to form structures reminiscent of barrels that are targeted by thalamocortical afferents, indicating that RORβ may regulate cell–cell interactions and axon-targeting (Jabaudon *et al.* 2012). Brain-region-specific knockout and overexpression of *Rorb* have been shown to influence the abundance of projections from the superior colliculus to the dorsal lateral geniculate nucleus and lateral posterior nucleus, further supporting a role for RORβ in neural target selection (Byun *et al.* 2019). These functions are directly related to neural circuit formation and may partially explain the association of *RORβ* variants with human neurodevelopmental conditions.

There is strong evidence for the involvement of *RORβ* in epilepsy with multiple clinical reports describing familial and de novo variants, copy number variations, microdeletions, and balanced translocations in *RORβ* amongst epileptic patients (Boudry-Labis *et al.* 2013; Baglietto *et al.* 2014; Bartnik *et al.* 2014; Lal *et al.* 2015; Rudolf *et al.* 2016). In many cases, these are photosensitive epilepsy cases with comorbidities including behavioral and cognitive impairment, and less commonly autism with disruption of the sleep cycle (Rudolf *et al.* 2016; Coppola *et al.* 2019; Sadleir *et al.* 2020). A role for *RORβ* variants in bipolar disorder has been suggested previously (Mansour *et al.* 2009; McGrath *et al.* 2009; McCarthy *et al.* 2012; Lai *et al.* 2015). *RORβ* variants may also confer risk to autism spectrum disorder (Satterstrom *et al.* 2020). However, clinical reports with family histories demonstrating segregation of *RORβ* variants with either autism or bipolar disorders are lacking. A mechanistic link has been suggested where disrupting *RORβ* may alter thalamocortical axon guidance, as occurs in rodents, and influence thalamocortical circuitry associated with absence seizures (Sadleir *et al.* 2006; Jabaudon *et al.* 2012; Rudolf *et al.* 2016). However, additional mechanisms linking *RORβ* with these neurodevelopmental conditions have not been reported.

Here we describe 5 independent strains of mice with mutations in *Rorb* that arose spontaneously at The Jackson Laboratory. All mice display the characteristic high-stepping, duck-like gait phenotype of *Rorb-*knockout mice, but only 2 of the 5 mice display retinal abnormalities. We performed behavioral phenotyping on one strain, *Rorb^h5/h5^*, and found differences in social interaction, anxiety, and repetitive behaviors. Gene expression analyses of all 5 mutant strains revealed a shared gene expression signature associated with the unfolded protein response (UPR) in both the brain and spinal cord. We also performed region-specific gene expression analysis of brain hemi-sections, spinal cord, cortex, hippocampus, and cerebellum in *Rorb^h5/h5^* mice comparing to wild-type inbred DBA/1J, the background strain on which this *Rorb* mutation arose, to further characterize the impact of *Rorb* mutation across the CNS. Our results demonstrate behavioral phenotypes relevant to human neurodevelopmental disorders that will be of interest to mammalian geneticists and gene expression signatures that may suggest a druggable pathway, the UPR, through which *RORβ* variants may confer susceptibility to neurodevelopmental disorders.

## Materials and methods

### Identification of *Rorb* mutants and mapping

The Mouse Mutant Resource at The Jackson Laboratory monitors production colonies for the appearance of abnormal phenotypes. Through this program, 5 spontaneous high-stepping mutants were identified. These spontaneous mutants were bred to determine heritability, and each strain was established and maintained as a distinct line. We referred to these mice as “high steppers,” though the gait phenotype has also been described as “duck like,” and named the strains 1–5, in order of discovery. Official strain designations, the abbreviated names used throughout this manuscript, and the background strains on which each spontaneous mutation occurred are provided in Table 1. Mice were housed under standard conditions with 14:10-hour light/dark cycles and ad libitum access to food and water. All procedures were performed in accordance with The Guide on the Care and Use of Laboratory Animals and were approved by the Institutional Animal Care and Use Committee of the Jackson Laboratory. Affected mutants, presumed homozygous, from the first high-stepper strain, now *Rorb^h1/h1^*, were crossed with inbred strain BALB/cByJ to produce F1 mice that were presumed obligate heterozygotes. F1 mice were intercrossed to produce F2 mapping animals. A genome scan and later fine-mapping identified a 0.7 Mb region on chromosome 19 between markers D19MIT41 and D19MIT113 containing *Trpm6*, *D030056L22Rik*, *Carnmt1*, and *Rorb*.

**Table 1.** Strain designations, naming, and background strain of spontaneous *RORβ* mutants.

Official strain designation	Mutant allele	Homozygous mutant	Background strain
C57BL/6J-*Rorb^hstp^/J*	h1	*Rorb^h1/h1^*	C57BL/6J
B6.Cg-*Tyr^c-2J^/Rorb^hstp-4J^*^/^GrsrJ	h2	*Rorb^h2/h2^*	B6(Cg)-Tyr^c-2J^/J
B6.Cg-*^Tyrc-2J^/Rorb^hstp-3J^*/GrsrJ	h3	*Rorb^h3/h3^*	B6(Cg)-Tyr^c-2J^/J
B6.Cg-*Rorb^hstp-2J^*/GrsrJ	h4	*Rorb^h4/h4^*	B6.129S7-*Il1r1^tm1/mx^*/J
DBA/1J-*Rorb^hstp-5J^*/Rwb	h5	*Rorb^h5/h5^*	DBA/1J

### Genotyping

Toe biopsies were obtained from mice during the first postnatal week and digested overnight in Proteinase K solution, which was then used for PCR reactions. Products were visualized with standard electrophoretic techniques (*Rorb^h1/h1^, Rorb^Cre^, Scnn1a^Cre^*, and *ROSA^tdT^*) or used for Sanger sequencing (*Rorb^h4/h4^, Rorb^h5/h5^*). Primers are as follows: *Rorb^h1/h1^* forward = AGGAGGAGGAATGGGAAGAA, reverse = TGTGAAGCCCTGCTATCCTT; *Rorb^h4/h4^,* forward *=* TCATGTGACAGGGGTCTGAA, reverse = GCCTTTGCATTGTCCAAAAA; *Rorb^h5/h5^,* forward *=* GGTAGTTTACTTGTAACAGGC, reverse = TTTCCAATCTGGGCAGCAGC; *Rorb^Cre^*: Forward = AACTTGCATGGGGAGAAGC, Reverse wild type (WT allele) = GTTCTCGTCCCCTTCATTTG; Reverse (*Cre* allele) = CCCTCACATTGCCAAAAGAC; *Scnn1a^Cre^* (generic *Cre*): Forward = GCATTACCGGTCGATGCAACGAGTG, Reverse = GAGTGAACGAACCTGGTCGAAATCA; *ROSA^tdT^* (WT allele): Forward = AAGGGAGCTGCAGTGGAGTA, Reverse = CCGAAAATCTGTGGGAAGTC; *ROSA^tdT^* (tdT allele): Forward = GGCATTAAAGCAGCGTATCC, Reverse = CTGTTCCTGTACGGCATGG.

### Behavior

Hot plate testing: Mice were brought into the testing room to acclimate for at least 10 min before testing. Animals were then individually placed on a hot plate set to 55°C; a Plexiglas cylinder was placed around the animal to keep them on the plate. The mouse was monitored for a maximum of 30 s and the time until the first hindpaw lick or flick was recorded. Control mice were a mix of *Rorb^+/+^* and *Rorb^+/h1^*.

Von Frey testing: Mice were removed from the main housing facility and brought to the testing room to habituate for no less than 30 min. Mice were then placed in the testing Plexiglas enclosure (9 cm L × 5 cm W × 5 cm H) on a wire-mesh floor with opaque sides and a clear front for observation. Mice were allowed to habituate to the testing chamber for 120 min before administration. Von Frey nylon monofilaments purchased from Stoelting (Cat # 18011) were used to test for mechanical sensitivity thresholds. Each monofilament was calibrated with a scale before testing. A series of 8 Von Frey fibers with logarithmically increasing stiffness (0.067–9.33 g) were used. Fibers were positioned perpendicular to the plantar surface of the hindpaw, alternating between left and right with a minimum of 5 min between responses. Enough pressure was applied to cause a slight bend in the filament and held for 6–8 s or until a response is elicited. Tests continued until a maximum of 9 determinations were made for each paw. Mechanical sensitivity thresholds were determined for each mouse over the course of 3 consecutive days. The threshold force required to elicit a response (median 50% paw withdrawal was determined using the up-down method (Chaplan *et al.* 1994). Upon completion of the day's trial, mice were returned to their home cage and monitored for up to 60 min before returning to the main housing room.

Open field activity assay: Mice were removed from the housing facility and allowed to habituate to the testing room for 60 min. Each mouse was then placed in the center of an arena (40 × 40 × 40 cm) and recorded for 60 min. Data are recorded via a sensitive infrared photobeam 3-dimensional grid system invisible to the mice. The automated system translates beam breaks into measurements such as distance traveled, rearing, circling, and repetitive behaviors (van den Buuse 2010).

Three-chamber social approach assay: Mice were removed from the housing facility and allowed to acclimate to the testing room for 60 min before testing. Each mouse was then habituated for 10 min in the middle chamber of an arena (40.5 × 60.0 × 22.0 cm) divided into 3 equal compartments with 1 cylindrical enclosure in each of the outer chambers. An unfamiliar adult mouse and a novel object were then placed in the 2 cylindrical enclosures in the outer chambers. The testing mouse was allowed to explore for 10 min. Time spent in each chamber and chamber entries were automatically recorded with video tracking (Noldus Ethovision) (Silverman *et al.* 2010). Mice that did not explore both chambers were excluded from analysis.

Grooming assay: mice were acclimated to the testing room for 60 min, and then each mouse was habituated to the testing chamber for 20 min. The mouse was misted with sterile water at room temperature during the testing phase. The experiment was video recorded, and a trained technician scored grooming behavior for the cumulative duration and the number of grooming bouts. Following the testing phase, mice were wiped with an absorbent paper towel if necessary before being returned to their home cage (Spruijt *et al.* 1992; Kalueff *et al.* 2007).

### Tissue collection

All tissues were collected following CO_2_ euthanasia. To collect retina, eyes were removed, and the lens and cornea were separated from the eye cup using fine scissors. For histology, the entire eye cup was fixed in 4% paraformaldehyde (PFA) for 4 hours at 4°C then immersed in 30% sucrose overnight at 4°C. Eye cups were then frozen in optimal cutting temperature compound (OCT) and cryosectioned. For qRT-PCR, retinas were gently removed from the eye cup, placed in RNALater (Life Technologies), and incubated overnight at 4°C. The tissue was then removed from RNALater and frozen at −80°C. Brain was removed and cut in half sagittally. For histology, one-half was then fixed overnight in 4% PFA at 4°C. Tissue was then mounted in an agarose block and sectioned at 100μm using a vibratome. For qRT-PCR, the half brain was cut into ∼6 pieces, placed in RNALater (Life Technologies), and incubated overnight at 4°C. Tissue was then removed from the RNALater and frozen at −80°C. For the spinal cord, the entire spinal column was removed and fixed for 24–48 h in 4% PFA at 4°C. The spinal cord was then removed from the vertebral column, immersed overnight in 30% sucrose, dissected to isolate the lumbar region, frozen in OCT, and cryosectioned at 20μm. Motor and sensory branches of the femoral nerve were removed and fixed overnight in 2% glutaraldehyde, and 2% paraformaldehyde, in 0.1 M cacodylate buffer at 4°C. Both nerve branches were embedded in plastic and 0.5-μm sections were cut and stained with toluidine blue.

### Histology and immunofluorescence

Hematoxylin/eosin staining was performed on retinal sections according to standard techniques. Antibodies used were as follows: 1:200 rabbit anti-S opsin (Millipore AB5407, RRID:AB_177457), 1:250 rabbit anticalcitonin gene-related peptide (CGRP) (Millipore PC205L-100UL, RRID:AB_564312), 1:250 rat antimyelin basin protein (MBP) (Millipore MAB386, RRID:AB_94975); secondary antibodies were Alexa Fluor conjugates from Life Technologies and used at 1:500. FITC-Peanut agglutinin (FITC-PNA) (Sigma L7381) was used at 0.01 mg/ml and Isolectin GS-IB_4_, Alexa Fluor 647 conjugate (Life Technologies I32450) was used at 1:200. Antibodies were applied in phosphate-buffered saline (PBS) containing 0.5% TritonX-100 and 3% fetal bovine serum and incubated on sections overnight at 4°C. Fluorophore-conjugated proteins were applied to sections during incubation with a secondary antibody; both were diluted in PBS. Retinal sections were imaged at 40 × using a NanoZoomer 2.0HT (Hematoxylin/eosin staining) or a Zeiss Axio Imager (fluorescently labeled sections). Both vibratome sections and cryosections from the brain and spinal cord were imaged using a Leica SP5 confocal. Single confocal slices are shown.

### Axon counting and quantification

For axon counting and axon area measurement, images were captured using a Nikon Eclipse E600 microscope with a 40 × objective and Nomarski DIC optics. Automated quantification was performed as described in detail previously (Bogdanik *et al.* 2013). Briefly, with the ImageJ software, the Threshold function was used to highlight axoplasm only on whole nerve sections; the Analyze Particle function was then used to count the number of myelinated axons and their cross-sectional areas in each nerve.

### 5′ RACE

5′ RACE was performed following manufacturer's protocol (Invitrogen 18374-041) on total RNA harvested from WT and *Rorb^h1/h1^* brain. The *Rorb*-specific primer used for first-strand synthesis was ACGTGATGACTCGTAGTGGA. For PCR of the RACE product, the primer GCCACAAATTTTGCATGGTA was used with the included abridged anchor primer.

### ChIA-PET

Mouse embryos were harvested at E12.5 for neural progenitor harvest. Neural progenitors were plated in 6 well plates coated with poly-L-ornithine and grown in N2B27 media for 9 days or until confluent. Ten million cells were dual-crosslinked with 1.5 mM Ethylene glycol bis(succinimidyl succinate) (EGS #21565, Thermo Fisher) for 45 min followed by 1% formaldehyde (F8775, Sigma) for 10 min at room temperature and then quenched with 0.2 M Glycine (G8898, Sigma) for 5 min. The crosslinked cells were washed with PBS twice and lysed in 100ul 0.55% SDS with incubation at room temperature, 62°C and 37°C sequentially for 10 min each, which was followed by 37°C for 30 min with the addition of 50μl 10% Triton-X 100 to quench SDS and 37°C overnight with addition of 40μl AluI from New England Biolabs (NEB R0137L) total, 50μl 10× CutSmart buffer to fragmentize the chromatin. The pelleted digested nuclei were resuspended in 500μl A-tailing solution containing 50μl 10× CutSmart buffer, 10μl BSA (B9000S, NEB), 10μl 10 mM dATP (N0440S, NEB), 10μl Klenow (3′−5′ exo-) (M0202L, NEB), and 420μl H_2_O with 1 h incubation at room temperature and then subjected to proximity ligation by adding 200μl 5× ligation buffer (B6058S, NEB), 6μl biotinylated bridge linker (200 ng/μl), 10μl T4 DNA ligase (M0202L, NEB), and incubating at 16°C overnight. The ligated chromatins were then sheared by sonication and immunoprecipitated with anti-CCCTC-binding factor (anti-CTCF) (Active Motive #61311). The immunoprecipitated DNA tagmentation, biotin selection, library preparation, and sequencing were performed as described (Zhang *et al.* 2013; Tang *et al.* 2015).

ChIA-PET data were processed with ChIA-PET Utilities, a scalable re-implementation of ChIA-PET Tools (Lee *et al.* 2020). In brief, the sequencing adaptors were removed from the pair-end reads, the bridge linker sequences were identified and the tags flanking the linkers were extracted. Tags identified (≥16 bp) were mapped to 10 mm using Burrows-Wheeler Alignment algorithm (BWA) alignment (Li and Durbin 2009) according to their tag length. The uniquely mapped, non-redundant pair-end tags (PETs) were classified as either interchromosomal (left tags and right tags aligned to the different chromosomes), intra-chromosomal (left tags and right tags aligned to the same chromosomes with genomic span >8 kb), and self-ligation (left tags and right tags aligned to the same chromosomes with genomic span ≤8 kb) PETs. Interacting PETs (iPETs), the uniquely mapped, non-redundant PETs from both the inter- (left tags and right tags from different chromosomes) and intra-chromosomal (left tags and right tags with genomic span >8 kb) PETs, were extended by 500 bp which was the average length of the sheared chromatin fragments. Multiple iPETs overlapping at both ends were then clustered as iPET-2, 3, … (clusters with 2, 3, … iPETs) to represent their interaction strength. Peaks were called using Model-based Analysis of ChIP-Seq (MACS version 2.1.0.20151222) with *q* < 1E-8. The called peaks were used to determine interactions supported by the CTCF binding (interaction anchors must overlap at least 1 bp on a peak span).

### qRT-PCR

Retinal and brain tissue was thawed and homogenized in Trizol using a mechanical homogenizer and total RNA was extracted using the manufacturer's protocol (Life Technologies 15596). cDNA synthesis (Life Technologies 18080-400) was performed using 1 μg total RNA and a 50/50 mix of oligo dT and random hexamers. qPCR was then performed using 1 μl of the resulting cDNA in a 20 μl reaction using standard SYBR green reagents (Life Technologies 4309155) on a ViiA 7 Real-Time PCR System. Results were analyzed using the ΔΔCt method, normalizing to Glyceraldehyde-3-phosphate dehydrogenase (GAPDH) expression and the average of the 2 WT control groups. Data are presented as arbitrary units, calculated as 100 times the fold change. Primers used for qPCR were as follows: *Rorb1/Rorb2* common primer set: forward = AGGAACCGTTGCCAACACTG, reverse = GACATCCTCCCGAACTTTACAG; *Rorb1*-specific: forward = GGCTGGGAGCTTCATGACTA, reverse = ACGTGATGACTCCGTAGTGGA; *Rorb2*-specific: forward = CCAGCCCAAAACTAAAGCTG, reverse = ACGTGATGACTCCGTAGTGGA; *GAPDH*: forward = AGGTCGGTGTGAACGGATTTG, reverse = TGTAGACCATGTAGTTGAGGTCA.

### RNA sequencing

RNASeq was performed on whole brains cut sagittally and then snap-frozen in liquid nitrogen. Spinal cords were hydraulically extruded using ice-cold DEPC 1X PBS and a 5 mL syringe before being snap frozen in liquid nitrogen. Frozen tissue samples were transferred to −80°C for storage. Brains from *Rorb^h5/h5^* were cut sagittally. One half was snap frozen as with other genotypes, and a half was used for brain-region-specific dissection. The cortex, hippocampus, and cerebellum were dissected free and snap-frozen individually before transfer to −80°C. Total RNA was isolated using a NucleoMag RNA Kit (Macherey-Nagel) and the KingFisher Flex purification system (ThermoFisher). Frozen tissues were pulverized using a Bessman Tissue Pulverizer (Spectrum Chemical) and homogenized in MR1 buffer (Macherey-Nagel) using a gentleMACS dissociator (Miltenyi Biotec Inc). RNA concentration and quality were assessed using the Nanodrop 8000 spectrophotometer (Thermo Scientific) and the RNA ScreenTape Assay (Agilent Technologies).

Libraries were constructed using the KAPA mRNA HyperPrep Kit (Roche Sequencing and Life Science), according to the manufacturer's protocol. Briefly, the protocol entails isolation of polyA-containing mRNA using oligo-dT magnetic beads, RNA fragmentation, first- and second-strand cDNA synthesis, ligation of Illumina-specific adapters containing a unique barcode sequence for each library, and PCR amplification. The quality and concentration of the libraries were assessed using the D5000 ScreenTape (Agilent Technologies) and Qubit dsDNA HS Assay (ThermoFisher), respectively, according to the manufacturer’s instructions. Libraries were sequenced 150 bp paired-end on an Illumina NovaSeq 6000 using the S4 Reagent Kit v1.5.

A standard RNA-Seq pipeline comprising tools to perform read quality assessment, alignment, and variant calling was adapted from a public nf-core pipeline (v3.5) at The Jackson Laboratory (Ewels *et al.* 2020). The pipeline takes sequence reads for each sample as raw fastq files and outputs read counts. FastQC was used for quality checks and then Trim Galore! was used to remove adapters and sequences with low quality (Fred < 20). Sequence reads that passed the quality threshold were aligned to a mouse reference (GRCm38) using the Spliced Transcripts Aligned to a Reference tool (STAR v2.7) and gene expression estimates were determined using RNA-Seq by Expectation Maximization (RSEM v1.3) with default parameters.

### Differential expression analysis

The R package DESeq2 was used to perform internal normalization of count estimates from RSEM and to test for differential expression (Love *et al.* 2014). Samples were analyzed in one batch with a model incorporating genotype and CNS region to explore biological sources of variation across the dataset. Principal component analysis (PCA) was used to visually inspect the separation of samples in this analysis. PCA revealed large effects of background strain and a tissue effect. To control for these effects and prevent them from confounding our analyses of genotype effects we reanalyzed samples in a pairwise fashion (mutant vs control) for each strain and tissue type individually, which successfully reduced the effect of background strain and tissue type. Gene lists from pairwise comparisons between each mutant genotype and strain-matched wild-type mice were filtered using a significance cutoff adjusted for multiple comparisons in DESeq2 (adj. *P* < 0.05). Raw data were deposited into the Gene Expression Omnibus (GEO), accession number 229218, and differential expression data are included in Supplementary Table 1.

### Over-representation and overlap analyses

Lists of differentially expressed genes (DEGs) from each pairwise comparison between mutants and wild types, within tissue, were uploaded to MouseMine “list” query (Motenko *et al.* 2015). Gene ontology terms (biological process, cellular compartment, and molecular function) (Ashburner *et al.* 2000; Harris *et al.* 2004; Gene Ontology 2021), Reactome pathways (Jassal *et al.* 2020), and Mammalian Phenotype Ontology terms (Smith and Eppig 2012) were ranked by Holm–Bonferroni adj. *P* and exported in Supplementary Table 2. The top 5 ontologies ranked by adj. p or false discovery rate (FDR) were –log10 transformed and visualized as dot plots made with GGPlot2 for R statistical software in R Studio (Wickham 2016). Volcano plots of DEGs were created with the EnhancedVolcano R package (Blighe *et al*. 2018). Sets of DEGs that appear repeatedly across multiple contrasts, and processes over-represented in this gene set, were found using ExpressAnalyst interactive heatmap functionality (https://dev.expressanalyst.ca/ExpressAnalyst/).

### Statistics

Statistical analysis of all behavioral phenotyping data, qRT-PCR, and nerve histology was performed in GraphPad Prism 9. Analysis of RNASeq data was performed using the DESeq2 package in R statistical software with R Studio. For behavior, comparisons between 2 groups (such as mutant v. control) were performed with unpaired *t*-tests. Comparisons involving multiple groups were performed using two-way ANOVAs with appropriate corrections for multiple testing (Tukey's multiple comparisons test for comparing means, i.e. genotypes in qPCR; Sidak's multiple comparisons test for comparing predetermined sets of means, i.e. vertical activity in open field). Over-representation analysis was performed in MouseMine, and Holm–Bonferroni multiple comparisons testing was used to determine significance.

## Results

### Five strains of high-stepping mice have spontaneous mutations affecting *Rorb*

**Fig. 1.**
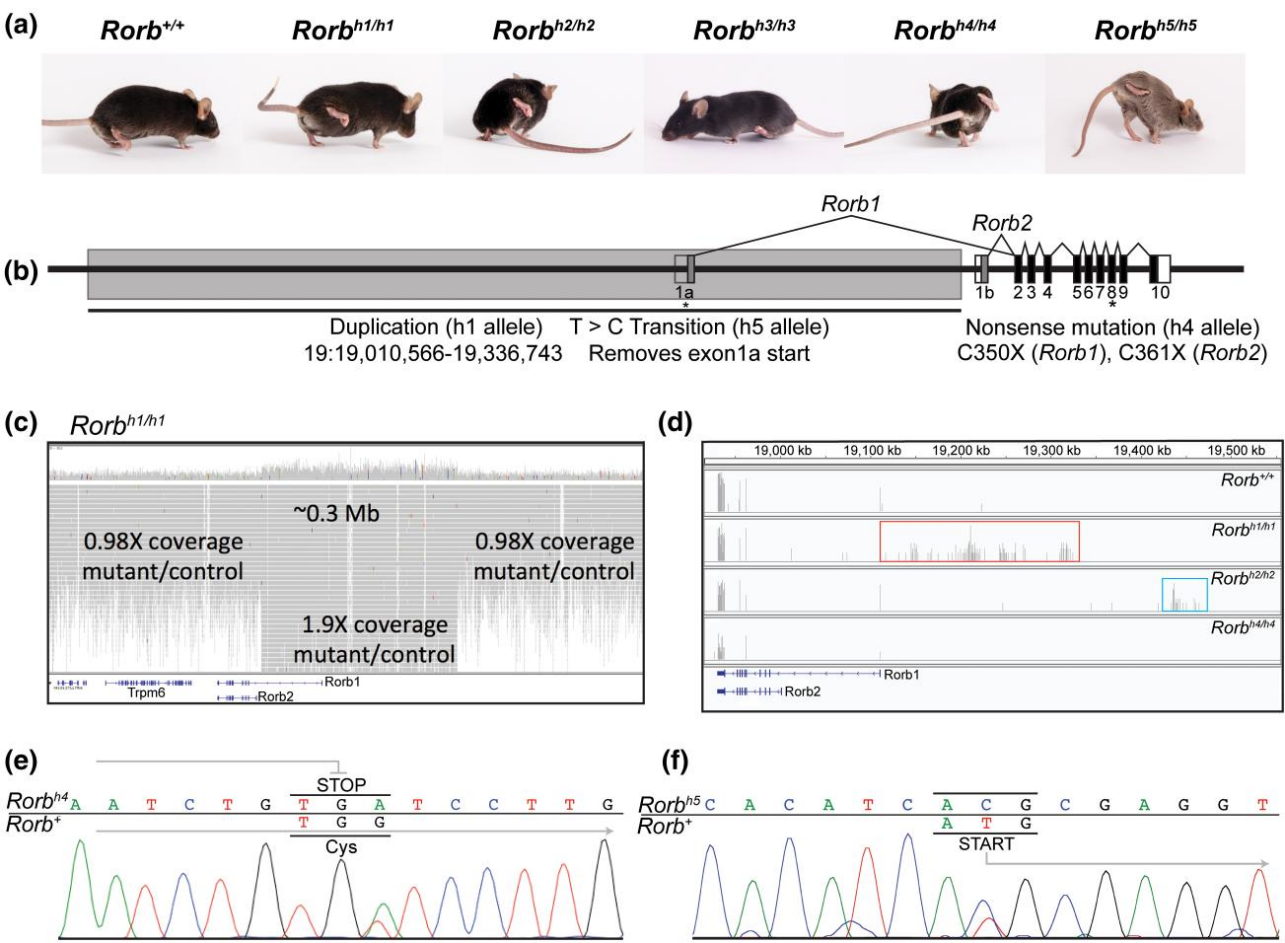
Spontaneous *Rorb* mutants display an abnormal gait phenotype. Spontaneous *Rorb* mutant strains exhibit hindlimb hyperflexion previously reported in *Rorb-*knockout mice. Note, although *Rorb^h2/h2^* arose on an albino B6 background the mouse pictured here was produced by intercrossing F1s of a complementation test and carries 2 copies of the h2 allele (a). The gait phenotype is caused by mutations in *Rorb*, which encodes 2 transcripts (*Rorb1* & *Rorb2)* with alternative first exons (1a and 1b indicated in dark gray) and 9 additional shared exons (2–10 shaded black). The causal mutations of *Rorb^h1/h1^, Rorb^h4/h4^,* and *Rorb^h5/h5^* are indicated in the diagram. The gene model is inverted from reverse strand orientation for readability (b). Whole genome sequencing identified a 326 kb duplication in *Rorb^h1/h1^* mice where a region on chromosome 19 upstream of *Rorb* shows 1.9X coverage compared to wild type (c). RNASeq alignments in the Integrative Genomics Viewer (IGV) show increased coverage upstream of *Rorb* in *Rorb^h1/h1^* mice and *Rorb^h2/h2^* mice (boxed regions) (d). Note the gene model in panels (c) and (d) reads right to left because *Rorb* is located on the negative strand of chromosome 19. Sanger sequencing reveals a G > A substitution that replaces a cysteine in exon 8 of *Rorb* with a premature stop codon in *Rorb^h4/h4^* mice (e). Sanger sequencing reveals a T > C substitution within the ATG start codon of exon 1a of *Rorb1* in *Rorb^h5/h5^* mice (f).

Between 2003 and 2020, 5 lines of spontaneous mutant mice with an overt high-stepping gait phenotype were identified in the production facility of The Jackson Laboratory (Fig. 1a; Supplementary Videos S1–6). These mice were referred to as “high-steppers,” and numbered one through 5 in order of discovery. We refer to them here as *Rorb^h1/h1^*, *Rorb^h2/h2^*, *Rorb^h3/h3^*, *Rorb^h4/h4^*, and *Rorb^h5/h5^*. In all cases, the inheritance pattern of the gait phenotype was consistent with a monogenic recessive mode of inheritance. We performed a genome scan and fine mapping with the first mutant, *Rorb^h1/h1^*, by crossing affected (presumed homozygous) mutants with BALB/cByJ mice to produce obligate heterozygotes (F1). These mice were then intercrossed for mapping, which identified a 0.7 Mb region on mouse Chromosome 19 between markers D19MIT41 and D19MIT113. This interval contains 4 protein-coding genes: *D030056L22Rik*, *Carnmt1*, *Trpm6*, and *Rorb.* The similarity between the gait phenotype of our mutants and previous descriptions of a duck-like gait in *Rorb-*knockout mice led us to suspect that *Rorb* was the causal gene (Andre, Conquet *et al.* 1998).

We performed Sanger sequencing of both *Rorb1* and *Rorb2* transcripts using genomic and cDNA, but no differences from C57BL/6J reference sequences were identified. For this reason, we included *Rorb^h1/h1^* mice in a large whole-genome sequencing project to identify spontaneous mutations (Fairfield *et al.* 2015). Whole-genome sequencing revealed a 326 kb duplication that includes the first exon specific to *Rorb1* and 5′ non-coding sequence but does not appear to include any exons of *Rorb2* (Fig. 1, b and c). Increased read depth was observed aligning upstream of *Rorb* in *Rorb^h1/h1^* mice by RNASeq, which was also observed in *Rorb^h2/h2^* mice, though the latter were not included in whole-genome sequencing and the specific causal mutation is unknown (Fig. 1d). The specific causal mutation also remains elusive in *Rorb^h3/h3^* mice, but complementation tests suggest that *Rorb* mutations underlie the gait phenotype in *Rorb^h2/h2^* and *Rorb^h3/h3^* mice (Table 2). Nonsense mutations were identified by Sanger sequencing in both *Rorb^h4/h4^* and *Rorb^h5/h5^* mice. A guanine to adenine substitution in exon 8 introduces a premature stop codon that is predicted to impact both *Rorb* isoforms in *Rorb^h4/h4^* mice (GRCm39: ENSMUST00000040153.15 Rorb-201, ENSMUST00000112832.8 Rorb-203) (Fig. 1e). In *Rorb^h5/h5^* mice, a thymine to cytosine substitution falls within the start codon of exon 1a of *Rorb1* (Fig. 1f). The next in-frame start codon of *Rorb1* is in exon 3, suggesting that the *Rorb^h5/h5^* start-loss variant likely disrupts the first *Rorb1* zinc finger domain encoded by exons 2 and 3 (GRCm39: ENSMUST00000112832.8 Rorb-203) (Cunningham *et al.* 2022).

**Table 2.** Complementation tests between spontaneous *Rorb* mutant strains.

Cross	# High-stepping F1s	Total born	Result
*Rorb^+/h4^* X *Rorb^+/h3^*	2	31	Weak/failure to complement
*Rorb^+/h2^* X *Rorb^+/h3^*	0	40	Complement
*Rorb^+/h1^* X *Rorb^+/h2^*	3	12	Weak/failure to complement
*Rorb^+/h1^* X *Rorb^+/h3^*	0	13	Complement

### Further analysis of *Rorb* expression and regulation in *Rorb^h1/h1^* mutants

*Rorb^h1/h1^* mice were the first high-stepping mutant identified by our group in 2003, and additional experiments were performed to characterize this strain and to better understand the mechanisms that disrupt fluid gait. We examined the expression of *Rorb* in cortical layer IV using a tdTomato reporter driven by either a *Rorb^Cre^* or *Scnn1a^Cre^* line (Harris *et al.* 2014). The *Rorb^Cre^* line was generated by insertion of an internal ribosome entry site cre (IRES-Cre) downstream of the translational stop. Homozygous *Rorb^Cre/Cre^* mice were reported to have an abnormal gait with high-stepping, and although we did not observe a gait phenotype in homozygous *Rorb^Cre/Cre^* mice, compound heterozygous *Rorb^Cre/h1^* mice did display a gait phenotype in our hands. We crossed *Rorb^+/h1^* mice with both *Rorb^Cre^* and *Scnn1a^Cre^* mice to drive tdTomato expression in RORβ+ and SCNN1A+ neurons of cortical layer IV, respectively. We found that tdTomato expression in cortical layer IV was uniform between *Rorb^+/Cre^* and *Rorb^Cre/h1^* F1s (Supplementary Fig. 1, a and b). tdTomato expression was markedly reduced in *Rorb^h1/h1^* compared to *Rorb^+/+^* F1 mice when crossed with the *Scnn1a^Cre^* line (Supplementary Fig. 1, c and d).

We also examined tdTomato expression driven by *Rorb^Cre^* in the spinal cord. We found reporter expression in the dorsal horn and scattered cells within the white matter (Supplementary Fig. 2, a and b). Co-labeling with CGRP and Isolectin-b4 revealed expression of *Rorb* in Isolectin-b4-, but not CGRP-, positive cells suggesting that some *Rorb-*positive cells may be part of the nonpeptidergic nociception pathway (Supplementary Fig. 2, c and d). We also observed co-labeling of tdTomato and MBP indicating that some *Rorb-*positive cells in the spinal cord are oligodendrocytes (Supplementary Fig. 2, e and f). Labeling was similar between *Rorb^+/Cre^* and *Rorb^Cre/h1^* mice and consistent with previous publications demonstrating the localization of RORβ+ neurons in the spinal cord (Koch *et al.* 2017). We next asked whether femoral nerves were affected in *Rorb^h1/h1^* mice. We found no differences in the number of axons in either motor or sensory branches of femoral nerve, but we did detect a modest significant decrease in the average area of the motor nerve branch in *Rorb^h1/h1^* compared to controls (Supplementary Fig. 3, a and c). We did not find any significant differences between *Rorb^h1/h1^* mice and wild-type controls on either the hot plate test of thermal nociception or the von Frey test of mechanosensation (Supplementary Fig. 3, d and e).

To gain further insight into how the *Rorb^h1/h1^* duplication impacts transcript expression and regulation, we performed ChIA-PET using neural progenitor cells of the developing brain at E12.5. We mapped CTCF-mediated chromatin interactions across the duplication and found that chromatin interactions were missing and rearranged in *Rorb^h1/h1^* progenitors compared to wild type (Supplementary Fig. 4a). One missing loop in *Rorb^h1/h1^* overlaps a strong distal enhancer specific to the developing forebrain at E14.5–16.5. This may suggest that the duplication disrupts spatiotemporal regulation of *Rorb* during development. To examine if the duplication impacted the size or produced an abnormal 5′ end of *Rorb* transcripts, we performed 5′-RACE (Supplementary Fig. 4b). We found that normal *Rorb1* transcript is produced in *Rorb^h1/h1^* mice, and no other major species were detected with this approach.

### Retinal abnormality occurs in only two of the *Rorb* mutant strains

**Fig. 2.**
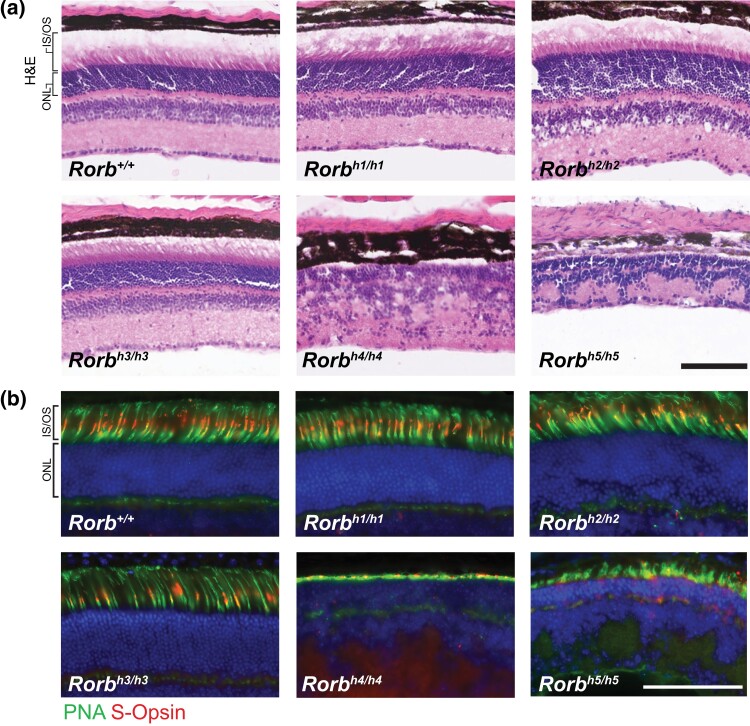
Retina histology reveals a structural abnormality in *Rorb^h4/h4^* and *Rorb^h5/h5^* mice. Representative images of hematoxylin and eosin staining of retina sections reveal no discernable difference in morphology between *Rorb^h1/h1^, Rorb^h2/h2^, Rorb^h3/h3^,* and wild-type littermates. The retinas of *Rorb^h4/h4^* and *Rorb^h5/h5^* mice lack the clearly defined layers of other genotypes (a). Representative images of retina sections stained with FITC-PNA and anti-S-opsin reveal intact photoreceptor inner and outer segments in *Rorb^h1/h1^*, *Rorb^h2/h2^*, *Rorb^h3/h3^*, and wild-type controls. Cone photoreceptor inner and outer segments are thinner in *Rorb^h4/h4^* and *Rorb^h5/h5^* mice (b). Scalebars = 100μm, *n* = 3/genotype at 6 weeks of age.

Postnatal retinal degeneration and developmental deficits have been previously reported in *Rorb*-deficient rats and mice (Jia *et al.* 2009; Liu *et al.* 2013; Fu *et al.* 2014). We examined retinal histology in the spontaneous *Rorb* mutants using hematoxylin/eosin staining to visualize gross morphology (Fig. 2a). *Rorb^h1/h1^*, *Rorb^h2/h2^*, and *Rorb^h3/h3^* mice have retinas that are morphologically indistinguishable from wild-type animals. *Rorb^h4/h4^* and *Rorb^h5/h5^* mice have highly disorganized retina without clear separation between layers in H&E staining. Both mutant strains appear to lack the inner and outer segments of photoreceptors. We used peanut agglutinin (PNA) and an anti-S-opsin antibody to visualize all cones and S-opsin positive cones, respectively (Fig. 2b). Again, *Rorb^h1/h1^*, *Rorb^h2/h2^*, and *Rorb^h3/h3^* animals had normal-appearing retinas while *Rorb^h4/h4^* and *Rorb^h5/h5^* animals exhibited a dramatically thinner layer of inner and outer segments from cones stained with PNA. S-opsin labeling was also substantially reduced in *Rorb^h4/h4^* and *Rorb^h5/h5^* mice compared to wild-type mice.

### *Rorb* transcript expression patterns in the brain and retina differ across strains

**Fig. 3.**
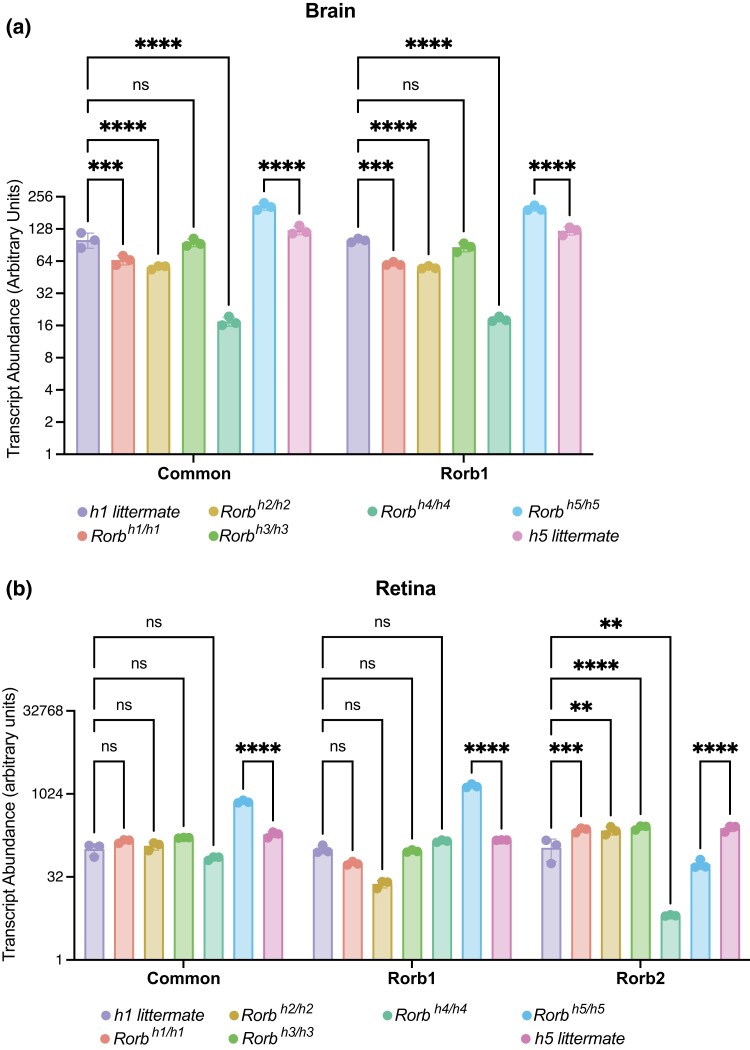
Expression of *Rorb1* and *Rorb2* transcripts in the brain and retina of *Rorb* mutant strains. Quantitative RT-PCR was performed on total RNA from brain (a) and retina (b) of all 5 spontaneous *Rorb* mutants with both C57BL/6J and DBA/1J control littermates (*n* = 3/genotype). *Rorb1-*specific, *Rorb2*-specific, and a common primer set were used to examine expression of transcripts encoding each *Rorb* isoform. Results are normalized to expression of *Gapdh* and the average of the 2 littermate control groups. Abundance is given as arbitrary units (equal to 100× the fold change) and shown as mean ± standard deviation on a log scale Y axis. Results are presented from mice aged 8 weeks. Data were analyzed with a two-way ANOVA with Tukey's multiple comparisons correction. *P < 0.05, **P < 0.01, ***P < 0.001, ****P < 0.0001, ns = not significant.

To assess the expression of each *Rorb* mRNA isoform in the brain and retina we designed 3 primer sets: one specific to the *Rorb1* transcript, another that amplifies exclusively the *Rorb2* isoform transcript, and a third common to both isoforms. In the brain, we found that *Rorb* expression assayed with the common primer set is significantly reduced in *Rorb^h1/h1^*, *Rorb^h2/h2^*, and *Rorb^h4/h4^* compared to strain-matched C57BL/6J littermate controls (Fig. 3a). *Rorb^h3/h3^* mice show no significant change in *Rorb* transcript levels. *Rorb1* expression follows the same pattern. *Rorb2* is not appreciably expressed in the brain. In the retina, we found that expression of *Rorb* amplicons from both common and *Rorb1* primers did not differ significantly between C57BL/6J controls and *Rorb^h1/h1^, Rorb^h2/h2^, Rorb^h3/h3^*, and *Rorb^h4/h4^* (Fig. 3b). *Rorb^h5/h5^* mice reverse this trend with elevated *Rorb* expression above strain-matched DBA/1J levels using common and *Rorb1*-specific primers in brain and retina. *Rorb2* expression was significantly increased above C57BL/6J levels in *Rorb^h1/h1^, Rorb^h2/h2^*, and *Rorb^h3/h3^* mice, while it was significantly lower in *Rorb^h4/h4^* mice. *Rorb^h5/h5^* mice also have significantly lower levels of *Rorb2* expression in the retina than DBA/1J controls providing a possible explanation for the shared retinal phenotype of *Rorb^h4/h4^* and *Rorb^h5/h5^* mice.

### Behavioral phenotypes in *Rorb^h5/h5^* mice

**Fig. 4.**
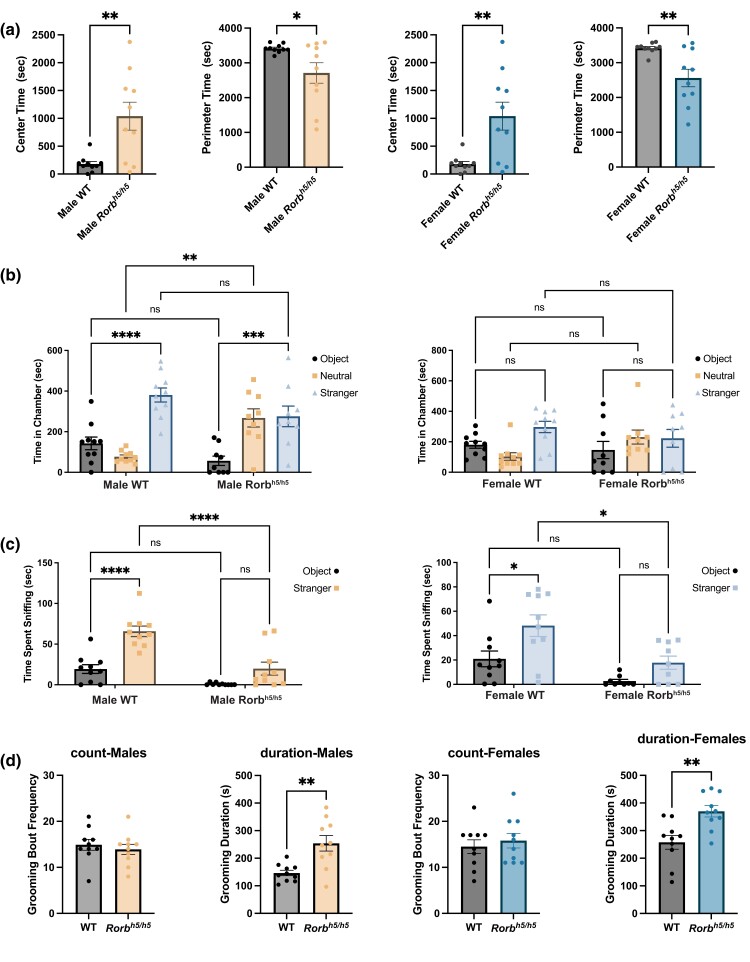
Open field, 3 chambers, and grooming behavior assays with *Rorb^h5/h5^* mice. Time spent in the open field arena center and arena perimeter is compared in male and female *Rorb*^h5/h5^ mice with strain-matched wild-type controls (*n* = 10/sex/genotype) (a). Three-chamber assay was performed with male and female *Rorb^h5/h5^* and littermate controls. The time spent in the same chamber as a novel object, a novel conspecific, or neither (neutral) is reported as a bar graph (b). Time spent sniffing the novel conspecific is reported for the 3-chamber assay (c). Sample size (*n* = 9–10/sex/genotype) (b and c). Grooming frequency and duration were assessed in *Rorb^h5/h5^* mice with strain-matched wild-type controls (*n* = 10/sex/genotype) (d). Unpaired *t*-test was performed for pairwise comparisons (a and d). Two-tailed ANOVA with Sidak's multiple comparisons test was performed for comparisons between genotypes with multiple conditions (b and c). Error bars indicate mean ± SEM. *P < 0.05, **P < 0.01, ***P < 0.001, ****P < 0.0001, ns = not significant.

Because of the reported involvement of *RORβ* variants in epilepsy, bipolar, and autism spectrum disorders, we chose to examine behavioral phenotypes related to cognition, sociability, and stereotypy in *Rorb^h5/h5^* mice. These mice have an identified start-loss mutation in *Rorb1*, the predominant *Rorb* isoform expressed in the brain. For all behavioral testing comparisons were performed against strain-matched DBA/1J wild-type mice. Open field testing was performed to assess general behavior and exploratory responses. Neither male nor female *Rorb^h5/h5^* mice showed significant differences in distance traveled over time or vertical activity (rearing); however, both male and female *Rorb^h5/h5^* mice spent more time in the arena center and less time on the arena perimeter than did wild-type controls (Fig. 4a; Supplementary Fig. 5a–d). Female wild-type controls travel significantly farther than *Rorb^h5/h5^* females overall, but no such difference was observed in male mice (Supplementary Fig. 5, e and f). We assessed social approach behavior using the 3-chamber assay. Female *Rorb^h5/h5^* mice showed no significant difference from wild-type mice in time spent investigating a novel object or a novel conspecific (Fig. 4b). Male *Rorb^h5/h5^* mice spent significantly more time in the neutral area (not investigating the novel object or conspecific), though the time spent investigating either the object or conspecific did not significantly differ from that spent by wild-type mice. Both male and female *Rorb^h5/h5^* mice spent significantly less time sniffing the novel conspecific than wild-type controls (Fig. 4c). Finally, we assessed whether *Rorb^h5/h5^* exhibits excessive spontaneous grooming. We found that neither male nor female *Rorb^h5/h5^* mice groomed more frequently than wild-type controls, but in both cases, grooming duration was significantly increased (Fig. 4d).

### RNASeq of brain and spinal cord in *Rorb* mutant strains

We performed RNASeq using brain and spinal cord samples from all 5 spontaneous mutants to better understand changes in gene expression and disordered pathophysiological processes. We selected the brain because of the emerging relationship between *RORβ* variants and epilepsy, and we selected spinal cord because the profound gait phenotype observed in all strains is caused by dysfunction of spinal interneurons (Koch *et al.* 2017). Further, we chose to examine gene expression in cortex, hippocampus, cerebellum, half brain, and spinal cord of *Rorb^h5/h5^* mice and strain-matched DBA/1J controls to gain a better understanding to how *Rorb1* start-loss affects different CNS regions, and which pathophysiological processes may explain the behavioral phenotypes of this strain. We began by performing an unbiased exploratory analysis of RNASeq counts data across all strains, mutants, and controls, with PCA to understand sources of variation between samples due to biological factors other than genotype (Supplementary Fig. 6a). This approach revealed that the genetic background on which a mutation occurred (C57BL/6J vs DBA/1J) was the greatest source of variation within the dataset. Tissue type was a second major source of variation between samples.

Therefore, we used pairwise comparisons between mutant mice and strain-matched wild-type mice, analyzing each tissue separately, for differential expression analyses. This approach controlled for strain and tissue effects. PCA on these more limited contrasts indicates that genotype (*Rorb* mutation carrier vs noncarrier) separates samples along the first and second principal component for most comparisons (Supplementary Fig. 6b). In some cases, separation by genotype is observed along the first and third or second and third principal components. Generally, this is due to sex having a greater effect than genotype (i.e. *Rorb^h1/h1^* vs C57BL/6J and *Rorb^h2/h2^* vs C57BL/6J, brain), or due to slight age differences at sample collection (∼3 days) in the *Rorb^h5/h5^*vs DBA/1J, hippocampus. However, in all cases, the effect of genotype is amongst the top principal components after controlling for genetic background and tissue type. For a simple common-sense check of RNASeq data we compared the expression of *Rorb* by RNASeq with qRT-PCR results and found the same expression pattern in the brain across strains (Supplementary Fig. 6c). Next, we performed differential gene expression analysis using DESeq2 (Love *et al.* 2014). Results are provided in Supplementary Table 1.

**Fig. 5.**
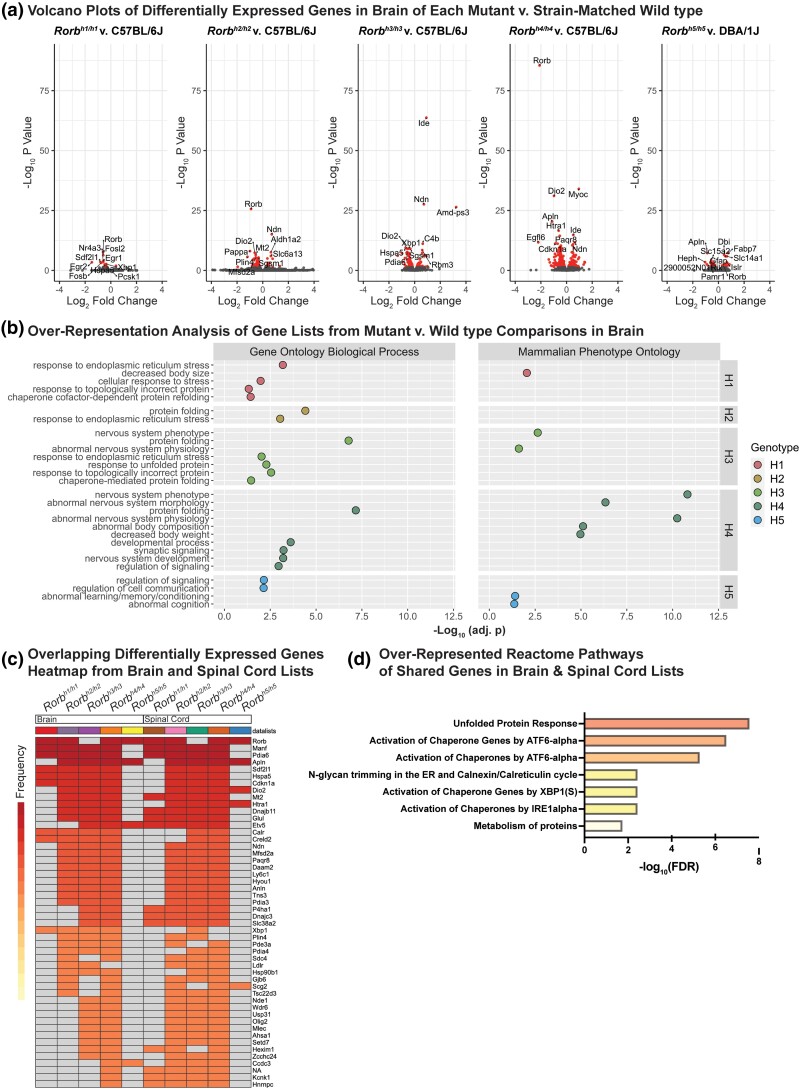
Analysis of gene expression and process over-representation in all *rorb* mutant strains vs strain-matched wild-type mice. Volcano plots from brain samples of each *Rorb* mutant strain contrasted with strain-matched wild-type controls. DEGs with adj. *P* < 0.05 are colored in red. The top 10 genes by adj. *P-*value is labeled, and *Rorb* is always labeled regardless of rank when its adj. *P* < 0.05 (A). The top 5 Gene Ontology biological process and Mammalian Phenotype Ontology terms ranked by Holm–Bonferroni *P-*value identified by over-representation analysis of differentially expressed gene lists from brain of each strain compared to strain-matched wild types are depicted as dot plots with –log_10_(adj. p) on the *x*-axis. Points are colored to indicate genotype (b). Genes that appear consistently differentially expressed in both brain and spinal cord of each mutant vs wild type comparison were identified by the Interactive Heatmap tool of ExpressAnalyst. Cells are shaded to indicate overlap frequency between contrasts (c). Reactome pathways that are over-represented amongst these shared genes identified with ExpressAnalyst are presented with –log_10_ transformed FDR values along the x-axis (D). All conditions (*n* = 3 mice, 7–9 weeks of age).

In general, gene expression changes are relatively subtle across strains. We chose to analyze those DEGs with an adj. *P* < 0.05. Comparison of *Rorb^h4/h4^* vs C57BL/6J mice identified the most DEGs of all comparisons in both brain and spinal cord. The number of DEGs for all strains and tissues is provided in Table 3. We visualized DEGs in brain and spinal cord for all genotypes using volcano plots (Fig. 5a; Supplementary Fig. 7a). *Rorb* falls amongst the top 10 DEGs ranked by adj. *P-*value in most pairwise comparisons. A notable exception is *Rorb^h3/h3^* vs C57BL/6J where *Rorb* differential expression does not pass the adj. *P* < 0.05 significance cutoff in either brain or spinal cord. *Rorb* is significantly differentially expressed in comparisons between *Rorb^h5/h5^* mice and DBA/1J mice for both brain and spinal cord, but it is not among the top 10 DEGs.

**Table 3.** DEGs for each comparison between mutant and wild type in brain and spinal cord.

Genotype comparison	Tissue	Genes adj. *P* < 0.05
*Rorb^h1/h1^* vs C57BL/6J	Brain	17
*Rorb^h1/h1^* vs C57BL/6J	Spinal cord	139
*Rorb^h2/h2^* vs C57BL/6J	Brain	83
*Rorb^h2/h2^* vs C57BL/6J	Spinal cord	526
*Rorb^h3/h3^* vs C57BL/6J	Brain	189
*Rorb^h3/h3^* vs C57BL/6J	Spinal cord	430
*Rorb^h4/h4^* vs C57BL/6J	Brain	443
*Rorb^h4/h4^* vs C57BL/6J	Spinal cord	559
*Rorb^h5/h5^* vs DBA/1J	Brain	62
*Rorb^h5/h5^* vs DBA/1J	Spinal cord	92
*Rorb^h5/h5^* vs DBA/1J	Cortex	59
*Rorb^h5/h5^* vs DBA/1J	Hippocampus	44
*Rorb^h5/h5^* vs DBA/1J	Cerebellum	42

We examined Gene Ontology Biological Processes and Mammalian Phenotype Ontologies that were significantly over-represented in our lists of DEGs from the brain (Fig. 5b). Annotations corresponding to endoplasmic reticulum stress and protein folding were common across comparisons when *Rorb^h1/h1^, Rorb^h2/h2^, Rorb^h3/h3^,* and *Rorb^h4/h4^* mice were compared to C57BL/6J mice. Interestingly, the *Rorb^h5/h5^* vs DBA/1J comparison gene list is not significantly associated with these annotations and, instead, has annotations related to abnormal learning and cognition. We examined over-represented processes using gene lists from the spinal cord of all *Rorb* mutants as well (Supplementary Fig. 7b). In this set, terms related to abnormal nervous system physiology and development are most common. All over-representation analysis results are provided in Supplementary Table 2.

Examination of volcano plots revealed that several DEGs other than *Rorb* appear frequently across different comparisons, which led us to search for a consistent gene expression signature of *Rorb* mutant strains in both the brain and spinal cord. We used ExpressAnalyst to find these overlapping genes and to identify Reactome pathways associated with them (Fig. 5c). Unsurprisingly, *Rorb* is the most shared differentially expressed gene. The entire list of shared DEGs was significantly associated with the UPR and several of its downstream activators such as ATF6, XBP1, and IRE1 (Fig. 5d).

### CNS region-specific differential expression in *Rorb^h5/h5^* vs DBA/1j

To better understand the impact of the *Rorb1* start-loss mutation in *Rorb^h5/h5^* mice on gene expression, and to identify processes associated with the behavioral phenotypes of these mice, we performed RNASeq with cortex, hippocampus, cerebellum, brain hemi-section, and spinal cord in comparison to these same tissues from DBA/1J strain-matched wild types. We performed differential expression analysis as before and visualized DEGs using volcano plots (Supplementary Fig. 8a). *Rorb* is among the top 10 most significant genes in brain hemi-sections, spinal cord, and cortex. While *Rorb* is also significantly differentially expressed in the cerebellum and hippocampus it does not rank in the top 10 genes. We performed over-representation analysis as before and found a variety of gene ontology process, compartment, and function terms private to each region (Supplementary Fig. 8b). In the cerebellum, gene lists were associated with Gene Ontology terms related to transporter activity and Reactome pathways involving small molecule transport. Gene lists from both cortex and hippocampus were associated with terms pertaining to the extracellular matrix, though additional pathways (many related to collagen synthesis and degradation) are found in the cortex. Gene expression in the spinal cord was associated with Gene Ontology and Reactome terms related to lipid biosynthesis and axon ensheathment while brain gene lists were associated with Gene Ontology terms such as cell periphery and signaling regulation with Reactome pathways related to behavior.

## Conclusions

We have identified 5 lines of spontaneous *Rorb* mutant mice, 3 where the causal mutation has been identified (*Rorb^h1/h1^*, *Rorb^h4/h4^*, and *Rorb^h5/h5^*) and 2 where the specific nature of the mutation has not been identified (*Rorb^h2/h2^* and *Rorb^h3/h3^*). The likelihood of *Rorb* involvement in *Rorb^h2/h2^* and *Rorb^h3/h3^* phenotypes is supported by the similarity of the gait phenotype between these mice and *Rorb*-knockout mice, complementation testing, and the pattern of *Rorb* expression by qRT-PCR. Notably, our complementation results indicate that both *Rorb^h2/h3^* and *Rorb^h1/h3^* complemented while *Rorb^h1/h2^* and *Rorb^h3/h4^* failed to complement. This mixed result may suggest that one or more of the alleles causes downregulation but not total ablation of *Rorb*, which could cause variability in the strength of complementation. RNASeq results showing increased read depth in the region upstream of *Rorb* in *Rorb^h2/h2^* mice, an alignment pattern similar to that produced by the duplication in *Rorb^h1/h1^* mice, further support this conclusion. The dysregulation of protein folding and ER stress in both mutant strains add additional supporting evidence. Protein levels across strains would provide additional useful information, but our attempts at western blots did not work. We remain unsure why spontaneous *Rorb* mutations seem to arise so frequently, but their distinctive gait phenotype makes them easy to identify, which may indicate ascertainment bias plays a role. Perhaps the locus is also prone to structural changes, since *Rorb^h1/h1^* and probably *Rorb^h2/h2^* fall in this category.

The regulation and expression pattern of *Rorb* was characterized more thoroughly in *Rorb^h1/h1^* mice because their discovery and testing occurred before the publication which elucidated the cellular and electrophysiological mechanism underlying the gait phenotype (Koch *et al.* 2017). We used *Rorb^Cre^* and *Scnn1a^Cre^* lines to drive tdTomato expression. Each Cre is specific to cell populations in cortical layer IV, and our imaging shows visible changes in fluorescence intensity in the *Scnn1a+* cell labeling but not *Rorb+* cell labeling in *Rorb^h1/h1^* mice. This likely reflects changes in cell type composition related to the role of *Rorb* in cell fate specification (Clark *et al.* 2020). In the spinal cord, our observation of co-labeling between tdTomato in *Rorb+* cells and Isolectin-b4, but not CGRP, suggests the involvement of some *Rorb-*expressing cells in the nonpeptidergic nociception pathway consistent with previous reports (Koch *et al.* 2017). ChIA-PET performed on *Rorb^h1/h1^* brains at E12.5 identified the loss of a local chromatin association with a strong distal enhancer that is specific to the developing forebrain. While more work is needed to experimentally validate this enhancer and to characterize the impact of this disrupted enhancer interaction on developmental expression of *Rorb*, it suggests that the 326 kb duplication present in *Rorb^h1/h1^* mice is likely to disrupt the spatiotemporal developmental regulation of this gene.

We show that *Rorb^h1/h1^*, *Rorb^h2/h2^*, and *Rorb^h3/h3^* mice have retinas that appear normal when labeled with PNA and anti-S-opsin while *Rorb^h4/h4^* and *Rorb^h5/h5^* mice exhibit highly disorganized retinas that lack inner and outer segments of cones and have reduced S-opsin labeling. The abnormal morphology of the retinas is reminiscent of retina from *Rorb*-knockout mice (Andre, Conquet *et al.* 1998; Chow *et al.* 1998; Srinivas *et al.* 2006; Jia *et al.* 2009). We suspect this is due to the reduction of *Rorb2* expression in retina of *Rorb^h4/h4^* and *Rorb^h5/h5^* mice compared to wild-type controls, evident in qRT-PCR data, but the mechanism by which loss of a start codon in *Rorb1* of *Rorb^h5/h5^* mice causes a decrease in *Rorb2* expression in unclear. *Rorb2* expression is increased significantly in *Rorb^h1/h1^* and *Rorb^h2/h2^* strains, though again the underlying mechanism is not clear. Interestingly, *Rorb^h5/h5^* mice exhibit an increase in *Rorb1* expression in both brain and retina that the other *Rorb* strains do not share. This could be explained by compensatory upregulation of *Rorb1* transcript in response to the absence of a fully functional protein product.

Behavioral phenotyping was performed in *Rorb^h5/h5^* mice because the start-loss mutation in these mice impacts the *Rorb1* isoform, which is predominant in brain, and likely produces a hypomorphic or loss of function allele. We chose assays that might reveal changes in social behavior, repetitive behaviors, and anxiety because of the associations between *RORβ* variants and neurodevelopmental conditions including epilepsy, bipolar, and autism spectrum disorders. In open-field testing, we found that both male and female *Rorb^h5/h5^* mice spent more time in the center of the arena, indicating decreased anxiety. Using the 3-chamber assay, we found that male, but not female, *Rorb^h5/h5^* mice exhibited a significant preference for the neutral area (no novel object or conspecific). Further, both male and female *Rorb^h5/h5^* mice spent significantly less time sniffing the novel conspecific. These findings may be interpreted as a decrease in sociability in *Rorb^h5/h5^* mice. Finally, *Rorb^h5/h5^* male and female mice exhibit longer grooming bouts than wild-type controls, though bouts were no more frequent. Both decreased sniffing and repetitive grooming have been reported in other mice that display autism-like behaviors (Peca *et al.* 2011; Kalueff *et al.* 2016). Similar behavioral phenotypes have been observed in mice with deletions in epilepsy-related genes (Kim *et al.* 2020). Based on our knowledge of RORβ function, it may be the case that *Rorb* mutations disrupt the formation of the neural circuitry that is involved in these mouse behaviors.

RNASeq data identified *Rorb* expression patterns that were consistent with qRT-PCR in brain across strains and showed that *Rorb* mutations in these mice produced gene expression changes that are relatively subtle compared to differences arising from strain and CNS region. *Rorb^h1/h1^* mice vs C57BL/6J mice had the fewest DEGs of all strains in both the brain and spinal cord, and *Rorb^h4/h4^* vs C57BL/6J mice showed the greatest number in both regions. This is not surprising considering that *Rorb^h4/h4^* is the only strain where we expect that the mutation impacts both transcripts. *Rorb* is significantly differentially expressed in contrasts between all strains and strain-matched wild types except *Rorb^h3/h3^* vs C57BL/6J, which agrees with our qRT-PCR result. It is possible we did not detect differential expression of *Rorb* in *Rorb^h3/h3^* vs C57BL/6J mice because only the *Rorb* isoform specific to the retina is altered in this strain. Interestingly, *Rorb^h5/h5^* mice show elevated expression levels of *Rorb* above DBA/1J wild types in both qRT-PCR and RNASeq data. This may be explained by the upregulation of *Rorb* expression to compensate for a nonfunctional *Rorb1* transcript in the *Rorb^h5/h5^* mice. Our over-representation analysis showed that gene lists from contrasts of *Rorb^h1/h1^, Rorb^h2/h2^, Rorb^h3/h3^,* and *Rorb^h4/h4^* brain to C57BL/6J brain were all associated with protein folding and endoplasmic reticulum stress. Interestingly, *Rorb^h5/h5^* vs DBA/1J mice did not show these annotations and instead produced gene lists associated with abnormal cognition. We do not understand the cellular basis for this difference, but it may involve the unique increase in *Rorb1* transcript expression in this strain.

Region-specific differential expression analysis of *Rorb^h5/h5^* vs DBA/1J mice produced gene lists with unique Gene Ontology annotations across the CNS reflecting the different gene expression programs regulated by RORβ Gene lists from contrasts involving spinal cord of all strains, while still frequently associated with terms related to the endoplasmic reticulum and protein folding as in the brain, are more often associated with abnormal nervous system physiology and development. These annotations are consistent with the known functions of RORβ in neural circuit formation (Nakagawa and O’Leary 2003; Jabaudon *et al.* 2012; Oishi *et al.* 2016; Moreno-Juan *et al.* 2017; Byun *et al.* 2019; Clark *et al.* 2020). Interestingly, we also found that the genes most frequently identified in all contrasts, regardless of genotype or tissue, are associated with the UPR. Endoplasmic reticulum stress and the UPR have been directly implicated in epileptogenesis, bipolar disorder, and a variety of other neurological disorders with overlapping comorbidities (Kas *et al.* 2014; Pfaffenseller *et al.* 2014; Fu *et al.* 2020; Terabayashi and Hashimoto 2021; Kumble *et al.* 2022; Madhamanchi *et al.* 2022). This finding raises the possibility that the UPR may be an important pathway for pathogenesis and treatment targeting patients with *RORβ*-associated epilepsy.

## Supplementary Material

jkad131_Supplementary_Data

## Data Availability

All data necessary for confirming the conclusions of the article are present within the article, figures, and tables. Supplementary Figs. 1–8 are available on FigShare. Supplemental files available at figshare (https://doi.org/10.25387/g3.23277272): Supplementary Table 1—DEGs contains lists of DEGs from DESeq2 analysis ranked by adjusted *P*-value. Raw RNASeq data are available on the Gene Expression Omnibus at accession number GSE229218. Supplementary Table 2—Over-representation analysis with MouseMine contains all over-represented terms identified using MouseMine ranked by Holm–Bonferroni adjusted *P*-value and separated based on ontology (Gene Ontology, Mammalian Phenotype Ontology, etc). Supplementary Videos S1–6 are footage of each *Rorb* mutant strain and a C57BL/6J mouse walking across level surfaces.
