# Supplementary material for: An allelic series of spontaneous Rorb mutant mice exhibit a gait phenotype, changes in retina morphology and behavior, and gene expression signatures associated with the unfolded protein response
Source: G3 (Bethesda). 2023 Jun 10;13(8):jkad131. doi: 10.1093/g3journal/jkad131 (PMC10411600; doi:10.1093/g3journal/jkad131)
Supplement: jkad131_Supplementary_Data [file jkad131_supplementary_data.zip › Figure S2.pdf]

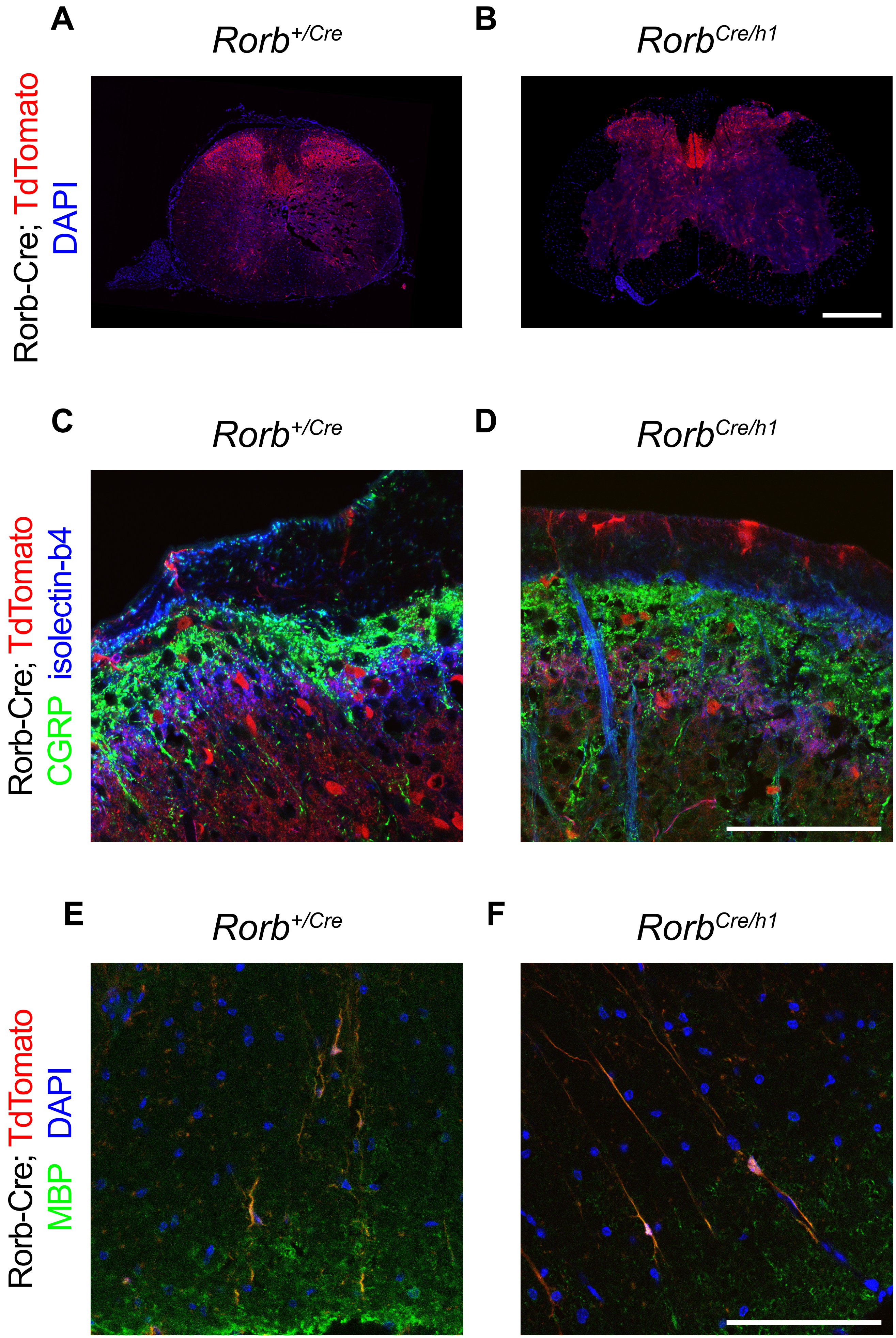
A
B
C
D
E
F
$Rorb^{+/Cre}$
$Rorb^{Cre/h1}$
Rorb-Cre; TdTomato DAPI
Rorb-Cre; TdTomato CGRP isolectin-b4
Rorb-Cre; TdTomato MBP DAPI
