# Supplementary material for: An allelic series of spontaneous Rorb mutant mice exhibit a gait phenotype, changes in retina morphology and behavior, and gene expression signatures associated with the unfolded protein response
Source: G3 (Bethesda). 2023 Jun 10;13(8):jkad131. doi: 10.1093/g3journal/jkad131 (PMC10411600; doi:10.1093/g3journal/jkad131)
Supplement: jkad131_Supplementary_Data [file jkad131_supplementary_data.zip › Figure S3.pdf]

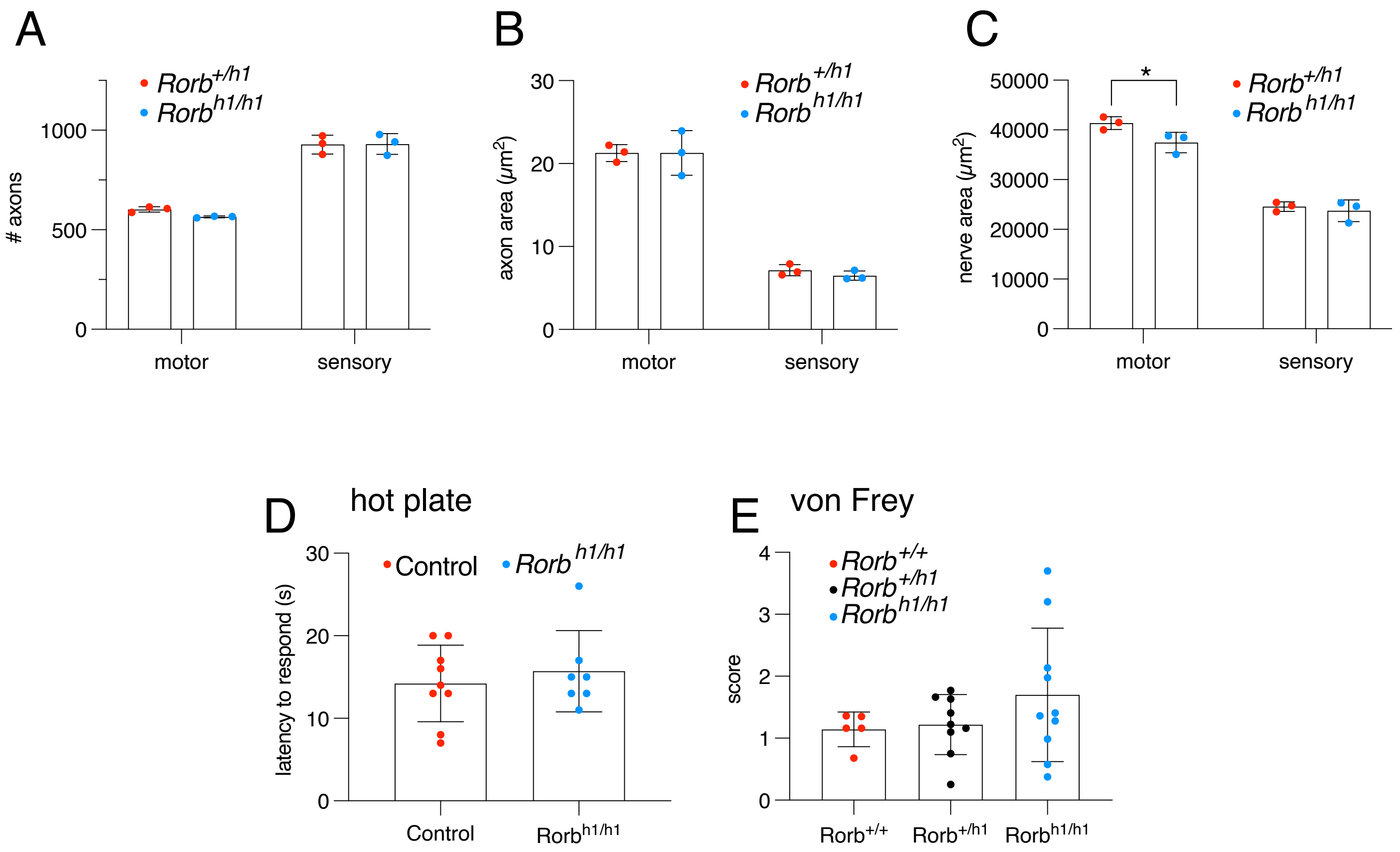
A
Rorb+/h1
Rorbh1/h1
axons
1000
500
0
motor
sensory
B
axon area (μm2)
30
20
10
0
Rorb+/h1
Rorbh1/h1
motor
sensory
C
nerve area (μm2)
50000
40000
30000
20000
10000
0
*
Rorb+/h1
Rorbh1/h1
motor
sensory
D
hot plate
Control
Rorbh1/h1
latency to respond (s)
30
20
10
0
Control
Rorbh1/h1
E
von Frey
Rorb+/+
Rorb+/h1
Rorbh1/h1
score
4
3
2
1
0
Rorb+/+
Rorb+/h1
Rorbh1/h1
