# Supplementary material for: An allelic series of spontaneous Rorb mutant mice exhibit a gait phenotype, changes in retina morphology and behavior, and gene expression signatures associated with the unfolded protein response
Source: G3 (Bethesda). 2023 Jun 10;13(8):jkad131. doi: 10.1093/g3journal/jkad131 (PMC10411600; doi:10.1093/g3journal/jkad131)
Supplement: jkad131_Supplementary_Data [file jkad131_supplementary_data.zip › Figure S4.pdf]

A

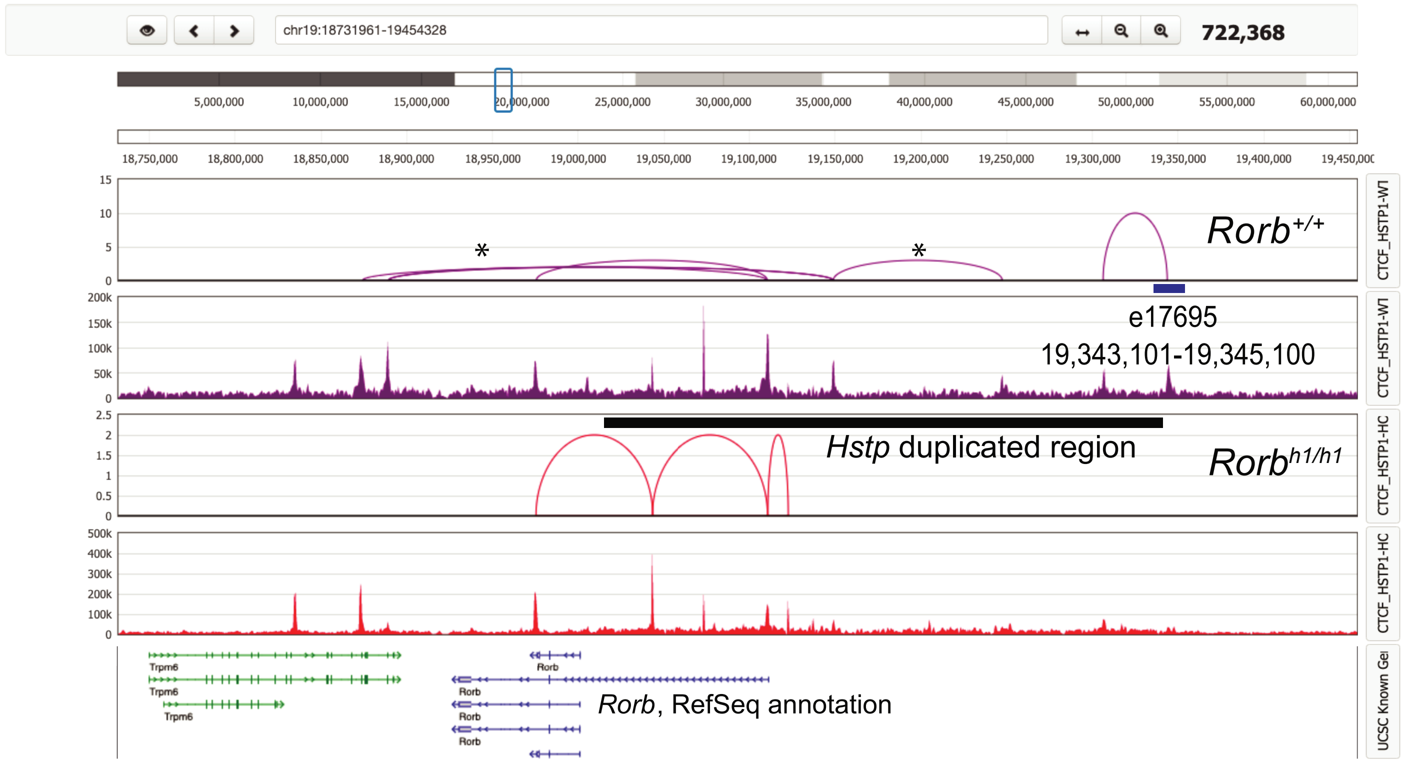
chr19:18731961-19454328
722,368
Rorb+/+
e17695
19,343,101-19,345,100
Hstp duplicated region
Rorbh1/h1
CTCF_HSTP1-W1
CTCF_HSTP1-HC
Rorb, RefSeq annotation
Trpm6
Rorb

B

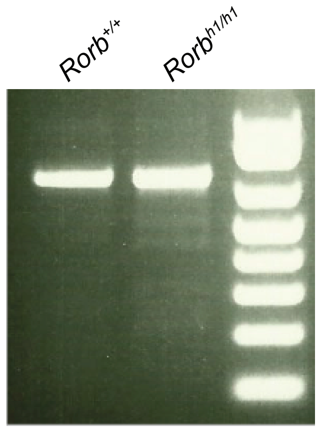
Rorb+/+
Rorbh1/h1
