# Supplementary material for: An allelic series of spontaneous Rorb mutant mice exhibit a gait phenotype, changes in retina morphology and behavior, and gene expression signatures associated with the unfolded protein response
Source: G3 (Bethesda). 2023 Jun 10;13(8):jkad131. doi: 10.1093/g3journal/jkad131 (PMC10411600; doi:10.1093/g3journal/jkad131)
Supplement: jkad131_Supplementary_Data [file jkad131_supplementary_data.zip › Figure S5.pdf]

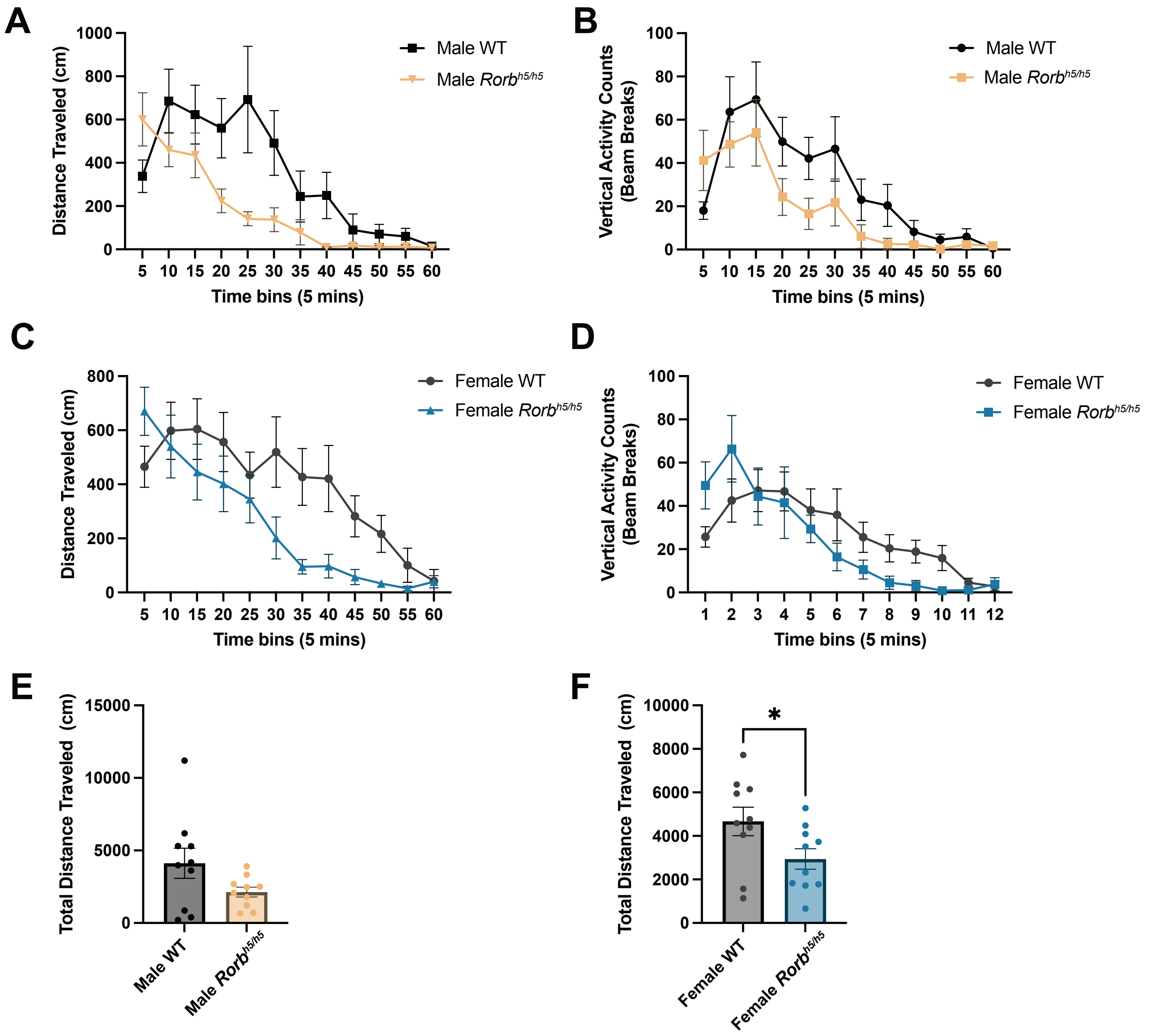
A
Distance Traveled (cm)
Time bins (5 mins)
Male WT
Male Rorb^h5/h5
B
Vertical Activity Counts (Beam Breaks)
Time bins (5 mins)
Male WT
Male Rorb^h5/h5
C
Distance Traveled (cm)
Time bins (5 mins)
Female WT
Female Rorb^h5/h5
D
Vertical Activity Counts (Beam Breaks)
Time bins (5 mins)
Female WT
Female Rorb^h5/h5
E
Total Distance Traveled (cm)
Male WT
Male Rorb^h5/h5
F
Total Distance Traveled (cm)
*
Female WT
Female Rorb^h5/h5
