# Supplementary material for: An allelic series of spontaneous Rorb mutant mice exhibit a gait phenotype, changes in retina morphology and behavior, and gene expression signatures associated with the unfolded protein response
Source: G3 (Bethesda). 2023 Jun 10;13(8):jkad131. doi: 10.1093/g3journal/jkad131 (PMC10411600; doi:10.1093/g3journal/jkad131)
Supplement: jkad131_Supplementary_Data [file jkad131_supplementary_data.zip › Figure S6.pdf]

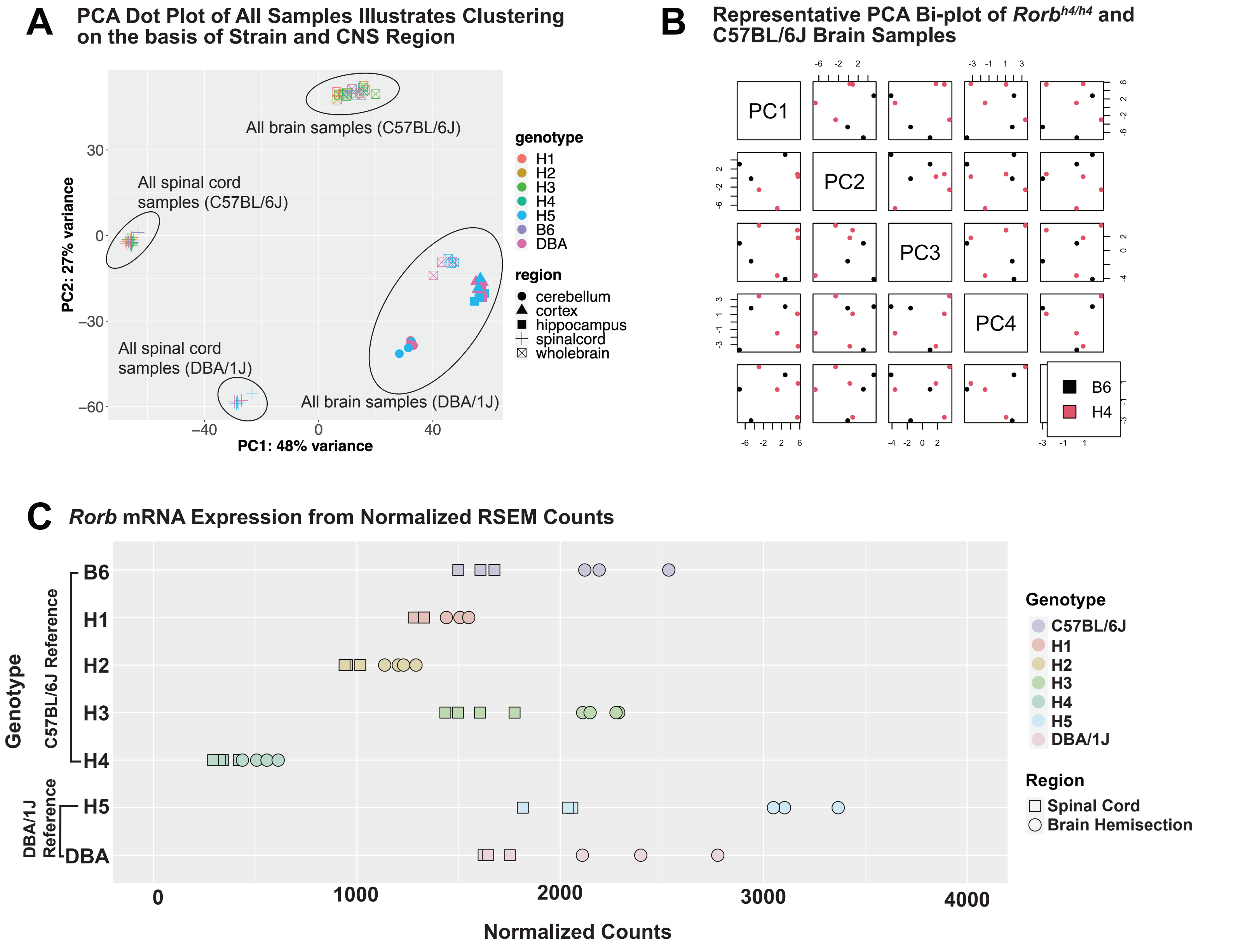
A
PCA Dot Plot of All Samples Illustrates Clustering on the basis of Strain and CNS Region
All brain samples (C57BL/6J)
All spinal cord samples (C57BL/6J)
All spinal cord samples (DBA/1J)
All brain samples (DBA/1J)
30
0
−30
−60
−40
0
40
PC2: 27% variance
PC1: 48% variance
genotype
H1
H2
H3
H4
H5
B6
DBA
region
cerebellum
cortex
hippocampus
spinalcord
wholebrain
B
Representative PCA Bi-plot of Rorb$^{h4/h4}$ and C57BL/6J Brain Samples
PC1
PC2
PC3
PC4
B6
H4
C
Rorb mRNA Expression from Normalized RSEM Counts
Genotype
C57BL/6J Reference
DBA/1J Reference
B6
H1
H2
H3
H4
H5
DBA
0
1000
2000
3000
4000
Normalized Counts
Genotype
C57BL/6J
H1
H2
H3
H4
H5
DBA/1J
Region
Spinal Cord
Brain Hemisection
