# Supplementary material for: An allelic series of spontaneous Rorb mutant mice exhibit a gait phenotype, changes in retina morphology and behavior, and gene expression signatures associated with the unfolded protein response
Source: G3 (Bethesda). 2023 Jun 10;13(8):jkad131. doi: 10.1093/g3journal/jkad131 (PMC10411600; doi:10.1093/g3journal/jkad131)
Supplement: jkad131_Supplementary_Data [file jkad131_supplementary_data.zip › Figure S7.pdf]

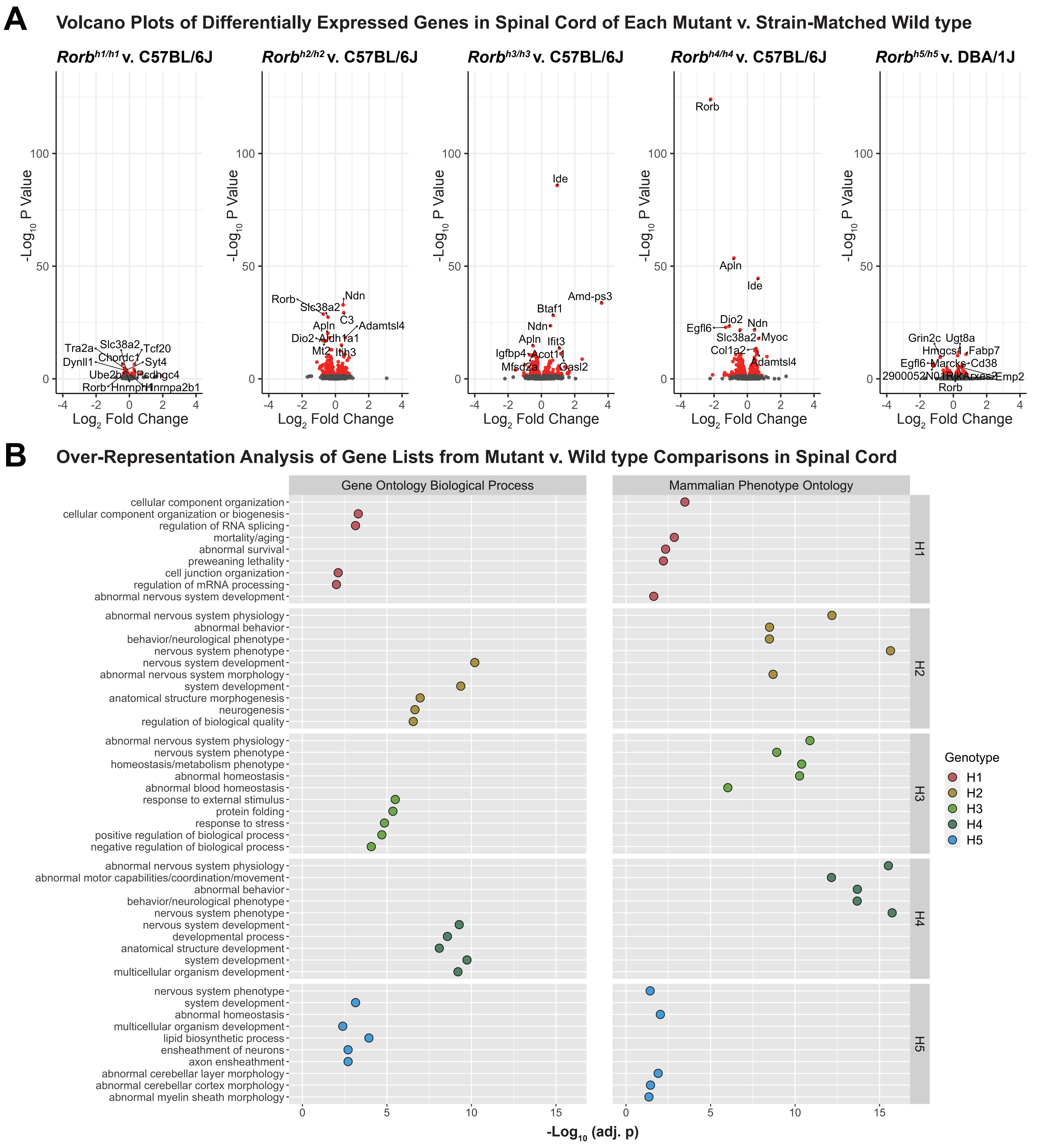
A
Volcano Plots of Differentially Expressed Genes in Spinal Cord of Each Mutant v. Strain-Matched Wild type
Rorb h1/h1 v. C57BL/6J
Rorb h2/h2 v. C57BL/6J
Rorb h3/h3 v. C57BL/6J
Rorb h4/h4 v. C57BL/6J
Rorb h5/h5 v. DBA/1J
-Log10 P Value
Log2 Fold Change
B
Over-Representation Analysis of Gene Lists from Mutant v. Wild type Comparisons in Spinal Cord
Gene Ontology Biological Process
Mammalian Phenotype Ontology
Genotype
H1
H2
H3
H4
H5
-Log10 (adj. p)
