# Supplementary material for: An allelic series of spontaneous Rorb mutant mice exhibit a gait phenotype, changes in retina morphology and behavior, and gene expression signatures associated with the unfolded protein response
Source: G3 (Bethesda). 2023 Jun 10;13(8):jkad131. doi: 10.1093/g3journal/jkad131 (PMC10411600; doi:10.1093/g3journal/jkad131)
Supplement: jkad131_Supplementary_Data [file jkad131_supplementary_data.zip › Figure S8.pdf]

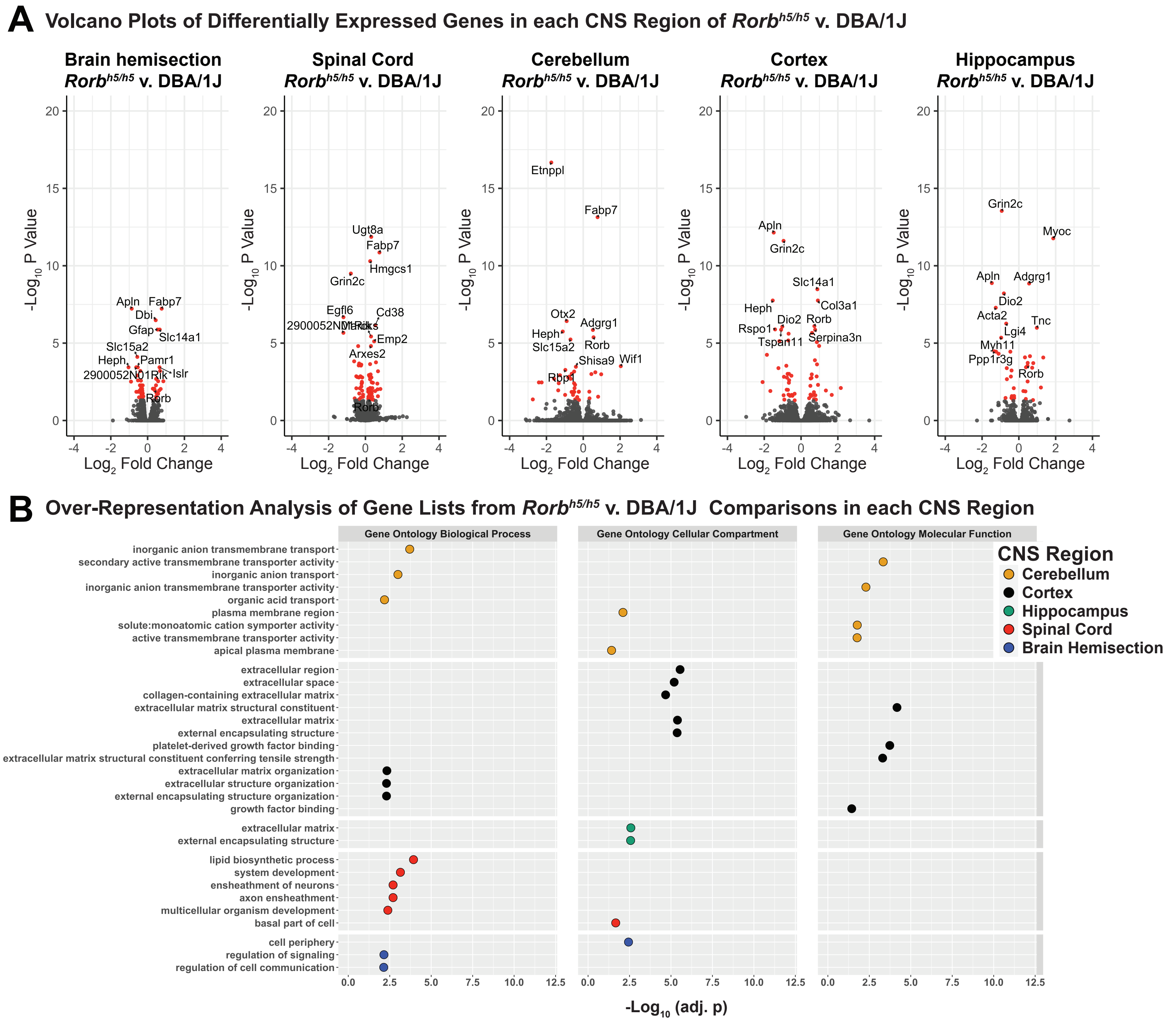
A Volcano Plots of Differentially Expressed Genes in each CNS Region of $Rorb^{h5/h5}$ v. DBA/1J
Brain hemisection $Rorb^{h5/h5}$ v. DBA/1J
Spinal Cord $Rorb^{h5/h5}$ v. DBA/1J
Cerebellum $Rorb^{h5/h5}$ v. DBA/1J
Cortex $Rorb^{h5/h5}$ v. DBA/1J
Hippocampus $Rorb^{h5/h5}$ v. DBA/1J
$-\text{Log}_{10}$ P Value
Log_2 Fold Change
Apln
Fabp7
Dbi
Gfap
Slc14a1
Slc15a2
Heph
Pamr1
Islr
Rorb
Ugt8a
Hmgcs1
Grin2c
Egfl6
Cd38
Emp2
Arxes2
Etnppl
Otx2
Adgrg1
Shisa9
Wif1
Rbp1
Col3a1
Dio2
Rspo1
Tspan11
Serpina3n
Myoc
Acta2
Tnc
Lgi4
Myh11
Ppp1r3g
B Over-Representation Analysis of Gene Lists from $Rorb^{h5/h5}$ v. DBA/1J Comparisons in each CNS Region
Gene Ontology Biological Process
Gene Ontology Cellular Compartment
Gene Ontology Molecular Function
inorganic anion transmembrane transport
secondary active transmembrane transporter activity
inorganic anion transport
inorganic anion transmembrane transporter activity
organic acid transport
plasma membrane region
solute:monoatomic cation symporter activity
active transmembrane transporter activity
apical plasma membrane
extracellular region
extracellular space
collagen-containing extracellular matrix
extracellular matrix structural constituent
extracellular matrix
external encapsulating structure
platelet-derived growth factor binding
extracellular matrix structural constituent conferring tensile strength
extracellular matrix organization
extracellular structure organization
external encapsulating structure organization
growth factor binding
extracellular matrix
external encapsulating structure
lipid biosynthetic process
system development
ensheathment of neurons
axon ensheathment
multicellular organism development
basal part of cell
cell periphery
regulation of signaling
regulation of cell communication
$-\text{Log}_{10}$ (adj. p)
CNS Region
Cerebellum
Cortex
Hippocampus
Spinal Cord
Brain Hemisection
