# Supplementary material for: An allelic series of spontaneous Rorb mutant mice exhibit a gait phenotype, changes in retina morphology and behavior, and gene expression signatures associated with the unfolded protein response
Source: G3 (Bethesda). 2023 Jun 10;13(8):jkad131. doi: 10.1093/g3journal/jkad131 (PMC10411600; doi:10.1093/g3journal/jkad131)
Supplement: jkad131_Supplementary_Data [file jkad131_supplementary_data.zip › Supplemental Material Legends.docx]

**SUPPLEMENTAL FIGURES**

**Figure S1 – tdTomato expression in *Rorb+* and *Scnn1a+* neurons of cortical layer IV.**  *Rorb^Cre^ -* driven tdTomato expression in coronal brain sections from *Rorb^+/Cre^* (A) and *Rorb^Cre/h1^* mice (B). Expression of tdTomato driven by *Scnn1a^Cre^* in *Rorb^+/+^* (C) and *Rorb^h1/h1^* (D) mice. Scale bars = 500 μm. Background images at 10X (A-D). Lower inset at 20X (A-B). Lower inset at 40X (C-D). All sections counterstained with DAPI.

**Figure S2 – tdTomato expression in *Rorb+* cells of the dorsal horn and co-labeling with Isolectin-b4 and MBP.**  Sections of spinal cord from the cervical enlargement reveal tdTomato expression in the dorsal horn of both *Rorb^+/Cre^* (A) and *Rorb^Cre/h1^* (B) mice. Co-labeling of tdTomato with CGRP (green) and Isolectin-b4 (blue) reveals colocalization between RORB and Isolectin-b4 but not CGRP in both *Rorb^+/Cre^* (C) and *Rorb^Cre/h1^* (D) mice. Co-labeling of tdTomato with MBP (green) reveals localization of some RORB+ cells within white matter of both *Rorb^+/Cre^* (E) and *Rorb^Cre/h1^* (F) mice. DAPI (blue) used in (A, B, E, F). Scale bar = 500 μm (A&B), scale bar = 100 μm (C-F). Sections imaged at 40X (A-B) or 63X (C-F).

**Figure S3 – *Rorb^h1/h1^* mice exhibit subtle changes in femoral nerve morphology but no change in response to thermal nociception or mechanosensation.** Heterozygous *Rorb^+/h1^* mice and *Rorb^h1/h1^* mice do not differ in the number of myelinated axons in motor or sensory branches of the femoral nerve (A). Axon area in femoral nerve motor and sensory branches does not differ between *Rorb^h1/h1^* mice and *Rorb^+/h1^* (B). *Rorb^+/h1^* mice have significantly larger nerve areas in the motor branch of the femoral nerve than *Rorb^h1/h1^* mice, though the difference is minor. There is no difference in nerve area in the sensory branch of the femoral nerve (C). Mice are at 9mo of age, n = 3 per genotype (A-C). Control littermates and *Rorb^h1/h1^* mice did not differ in response latency in the hot plate test of thermal nociception. Mice at 8mo of age, n = 7 Rorb^h1/h1^, 9 Rorb^+/+^ (D). *Rorb^+/+^*, *Rorb^+/h1^*, and *Rorb^h1/h1^* mice did not differ in the Von Frey test of mechanical sensation. Mice at 2mo of age n = 10 *Rorb^h1/h1^*, 9 *Rorb^+/h1^*, 5 *Rorb^+/+^* (E). Unpaired t-tests with mean ± standard deviation used for pairwise comparisons. One-way ANOVA with Tukey’s correction used for multiple comparisons reported as mean ± standard deviation. * p < 0.05.

**Figure S4 – *Rorb^h1/h1^* duplication impacts local chromatin associations but does not cause production of species that differ from wild-type C57BL/6J.** CTCF-interactions in neural progenitor cells from *Rorb^h1/h1^* and *Rorb^+/+^* brain at E12.5. Interactions within and around the duplicated region are missing or rearranged in *Rorb^h1/h1^* NPCs. The red peaks depict enrichment (normalized coverage) for DNA sequences bound by CTCF and the loops represent double-anchor factor bound supported chromatin loops. The duplicated region is shown in black, and a region containing a strong distal enhancer (e17695) specific to developing forebrain is indicated in blue (A). 5’ RACE from *Rorb^+/+^* and *Rorb^h1/h1^* does not indicate production of abnormal transcripts in *Rorb^h1/h1^* mice. Mice at 6 weeks, n=3 per genotype (B).

**Figure S5 – *Rorb^h5/h5^* mice show no difference in distance traveled over time or vertical activity compared to DBA/1J but females do exhibit significantly reduced total travel distance.** Male *Rorb^h5/h5^* do not differ significantly from wild-type controls in distance traveled over time (A) or vertical activity (B). Female *Rorb^h5/h5^* mice do not differ significantly from wild-type controls in distance traveled over time (C) or vertical activity (D). Male *Rorb^h5/h5^* mice do not differ from male *Rorb^+/+^* mice in total distance traveled (E). Female *Rorb^h5/h5^* mice travel significantly shorter distances than *Rorb^+/+^* females (F). N=10 per sex per genotype. Two-way ANOVA with Sidak’s correction for multiple comparisons (A-D). Unpaired t-test, * p < 0.05 (E&F). Mean ± SEM reported.

**Figure S6 – Exploratory Analysis of RNASeq in brain and spinal cord of all mutant strains.** Principal component analysis (PCA) of counts data from all samples indicates clustering of samples based on background strain (either C57BL/6J or DBA/1J) and CNS region. Shape indicates CNS region and color indicates genotype. Clusters are indicated with ellipses and labeled to indicate background strain and CNS region (A). A representative PCA bi-plot of *Rorb^h4/h4^* and C57BL/6J wild-type brain samples containing the first five principal components after controlling for strain and nervous system region. Separation of samples by genotype effect is evident along the first two components. Red dots are *Rorb^h4/h4^* samples and black dots represent C57BL/6J wild types (B). *Rorb* mRNA expression from DESeq2 normalized counts for each genotype and nervous system region presented as a dot plot. Normalized counts appear on the x-axis. Brackets on the y-axis indicate genotype and background strain. Color indicates genotype and shape indicates either brain or spinal cord (C). All conditions (n=3 mice, 8-9 weeks of age).

**Figure S7 - Gene expression analysis of spinal cord samples from each *Rorb* mutant and strain-matched wild-type mice.** Volcano plots created from differentially expressed gene lists of spinal cord samples of each *Rorb* mutant genotype compared to strain-matched wild-type mice. Differentially expressed genes with adj. p < 0.05 are colored in red. The top ten genes by adj. p value are labeled, and *Rorb* is always labeled regardless of rank when its adj. p < 0.05 (A). The top 5 Gene Ontology Biological Processes and Mammalian Phenotype Ontology terms ranked by Holm-Bonferroni p value in over-representation analysis are reported as dot plots with -log_10_ (adj. p) on the x-axis. Dot color indicates genotype (B). All conditions: n=3 mice, 7-9 weeks of age.

**Figure S8 – CNS Region-specific RNASeq in *Rorb^h5/h5^* mutants versus DBA/1J wild types.**  Volcano plots of differentially expressed genes from nervous system region-specific samples of *Rorb^h5/h5^* mice compared to DBA/1J wild types. Differentially expressed genes with adj. p < 0.05 are colored red. The top ten genes by adj. p value are labeled, and *Rorb* is always labeled regardless of rank when its adj. p < 0.05 (A). The top five Gene Ontology Biological Process, Cellular Component, and Molecular Function terms ranked by Holm-Bonferroni p value for each nervous system region are depicted as dot plots with -log_10_(adj. p) on the x-axis. Points are colored to indicate nervous system region (B). All conditions (n=3 mice, 7-9 weeks of age).

**SUPPLEMENTAL MATERIALS**

**Table S1 – Differentially Expressed Genes.**  Excel spreadsheet of differentially expressed genes from all comparisons, with each pairwise comparison as a separate tab. The mutant genotype and the strain-matched wild type are listed in the heading of each tab, as is the tissue type. Those genes indicated within boundary boxes have adj. p < 0.05 and were included in further analyses such as over-representation. Gene order is ranked by adj. p value.

**Table S2 – Over-representation Analysis with MouseMine.** Excel spreadsheet of significantly over-represented processes determined through analysis in MouseMine with lists of differentially expressed genes from all comparisons, with each pairwise comparison as a separate tab. The mutant genotype and the strain-matched wild type are listed in the heading of each tab, as is the tissue type. Significance was determined as Holm-Bonferroni p < 0.05. Processes are ranked within each ontology by order of adj. p value.

**Video S1 – Rorb h1*.*** A video recording of *Rorb^h1/h1^* mouse walking across a level surface.

**Video S2 – Rorb h2*.*** A video recording of *Rorb^h2/h2^* mouse walking across a level surface. **Video S3 – Rorb h3*.*** A video recording of *Rorb^h3/h3^* mouse walking across a level surface.

**Video S4 – Rorb h4*.*** A video recording of *Rorb^h4/h4^* mouse walking across a level surface. **Video S5 – Rorb h5*.*** A video recording of *Rorb^h5/h5^* mouse walking across a level surface.

**Video S6 – Rorb C57BL/6J*.*** A video recording of wild-type C57BL/6J mouse walking across a level surface.
